# Cryo-EM analysis of a feline coronavirus spike protein reveals a unique structure and camouflaging glycans

**DOI:** 10.1073/pnas.1908898117

**Published:** 2020-01-03

**Authors:** Tzu-Jing Yang, Yen-Chen Chang, Tzu-Ping Ko, Piotr Draczkowski, Yu-Chun Chien, Yuan-Chih Chang, Kuen-Phon Wu, Kay-Hooi Khoo, Hui-Wen Chang, Shang-Te Danny Hsu

**Affiliations:** ^a^Institute of Biological Chemistry, Academia Sinica, Taipei 11529, Taiwan;; ^b^Institute of Biochemical Sciences, National Taiwan University, Taipei 10617, Taiwan;; ^c^Graduate Institute of Molecular and Comparative Pathobiology, School of Veterinary Medicine, National Taiwan University, Taipei 10617, Taiwan;; ^d^Institute of Cellular and Organismic Biology, Academia Sinica, Taipei 11529, Taiwan

**Keywords:** cryoelectron microscopy, mass spectrometry, protein glycosylation, alphacoronavirus, feline infectious peritonitis virus

## Abstract

Feline infectious peritonitis virus (FIPV) is an alphacoronavirus that causes a nearly 100% mortality rate without effective treatment. Here we report a 3.3-Å cryoelectron microscopy (cryo-EM) structure of the serotype I FIPV spike (S) protein, which is responsible for host recognition and viral entry. Mass spectrometry provided site-specific compositions of densely distributed high-mannose and complex-type N*-*glycans that account for 1/4 of the total molecular mass; most of the N-glycans could be visualized by cryo-EM. Specifically, the N-glycans that wedge between 2 galectin-like domains within the S1 subunit of FIPV S protein result in a unique propeller-like conformation, underscoring the importance of glycosylation in maintaining protein structures. The cleavage site within the S2 subunit responsible for activation also showed distinct structural features and glycosylation. These structural insights provide a blueprint for a better molecular understanding of the pathogenesis of FIP.

## Significance

We report here a 3.3-Å cryo-EM structure of feline infectious peritonitis virus (FIPV) S protein derived from the serotype I FIPV UU4 strain. The near-atomic EM map enabled ab initio modeling of 27 out of the 33 experimentally verified high-mannose and complex-type N-glycans that mask most of the protein surface. We demonstrated the feasibility to directly visualize the core fucose of a complex-type glycan, which was independently cross-validated by glycopeptide mass spectrometry analyses. There exist 3 N-glycans that wedge between 2 galectin-like domains within the S1 subunit of FIPV-UU4 S protein, resulting in a propeller-like conformation unique to all reported CoV S proteins. The results highlight a structural role of glycosylation in maintaining complex protein structures.

Coronaviruses (CoVs) are enveloped viruses with single-stranded positive-sensed RNA. They are major infectious pathogens for a myriad of mammals and birds (1). Interspecies transmission due to genetic mutations in CoVs are responsible for life-threatening pandemics, such as severe acute respiratory syndrome-related CoV (SARS-CoV) in 2003 and Middle East respiratory syndrome-related CoV (MERS-CoV) in 2012 (1–3), resulting in detrimental economic and societal impacts. Sporadic CoV outbreaks further cause animal endemics and major economic losses (4–6). Crucially, there is a constant risk of zoonotic outbreaks that argues for a better molecular understanding of CoV-associated pathogenesis (7, 8).

First reported 4 decades ago, feline infectious peritonitis (FIP) is one of the most fatal infectious diseases in cats, particularly kittens (9). The disease results from infection by a feline CoV (FCoV), feline infectious peritonitis virus (FIPV) (10, 11). There is no effective treatment and the fatality rate is essentially 100% (10, 11). FCoV can be serologically categorized into 2 types: serotypes I and II. Serotype I FCoV is the most epidemiologically prevalent, contributing to more than 70% of circulating virus isolates worldwide (12). Progress in FIP research has been limited not least because of difficulties in isolating and propagating serotype I FCoV in vitro for mechanistic studies on pathogenesis at the molecular level, which are urgently needed for the development of vaccines and therapeutics (2, 9, 13, 14).

CoVs use their spike (S) proteins for host recognition and subsequent membrane fusion to introduce their viral genomes into the host for replication. Preventing CoV infection by blocking S-protein binding to host receptors therefore represents the first line of defense. CoV S proteins consist of 2 functional units, the S1 and S2 subunits, which are responsible for cell attachment and membrane fusion, respectively (1, 2, 15). Mutations in the receptor-binding motifs (RBMs) or cleavage sites of CoV S proteins can lead to zoonotic spillover and alteration of cell/tissue tropism, as exemplified by SARS and MERS (1, 16).

To better understand the molecular basis of FIPV, we constructed a trimeric S glycoprotein of a serotype I FIPV UU4 strain and overexpressed it in a mammalian cell line, as opposed to insect cell lines that were used in previous structural studies on CoV S proteins (3, 17–21). This ensured a better resemblance of the posttranslationally modified glycosylation patterns on the S protein, the chemical structures of which were determined by mass spectroscopy (MS). Cryoelectron microscopy (cryo-EM) single-particle reconstruction was employed to determine the 3-dimensional (3D) structure of FIPV-UU4 S protein to a resolution of 3.3 Å, enabling ab initio model building of not only the protein but also most of the N-glycan structures. The result enabled direct visualization of the camouflaging N-glycans on the surface of FIPV-UU4 S protein. Nearly 1/3 of the observed N-linked glycans correspond to the complex type, which has not been studied in such detail in previously reported CoV S proteins (3, 17–21). Our cryo-EM structure of FIPV-UU4 S protein therefore serves as a starting point for further studies on virus–host interactions and development of better vaccines and therapeutics for the intervention of FIP.

## Results

### Structure of FIPV-UU4 S Protein.

**Fig. 1.**
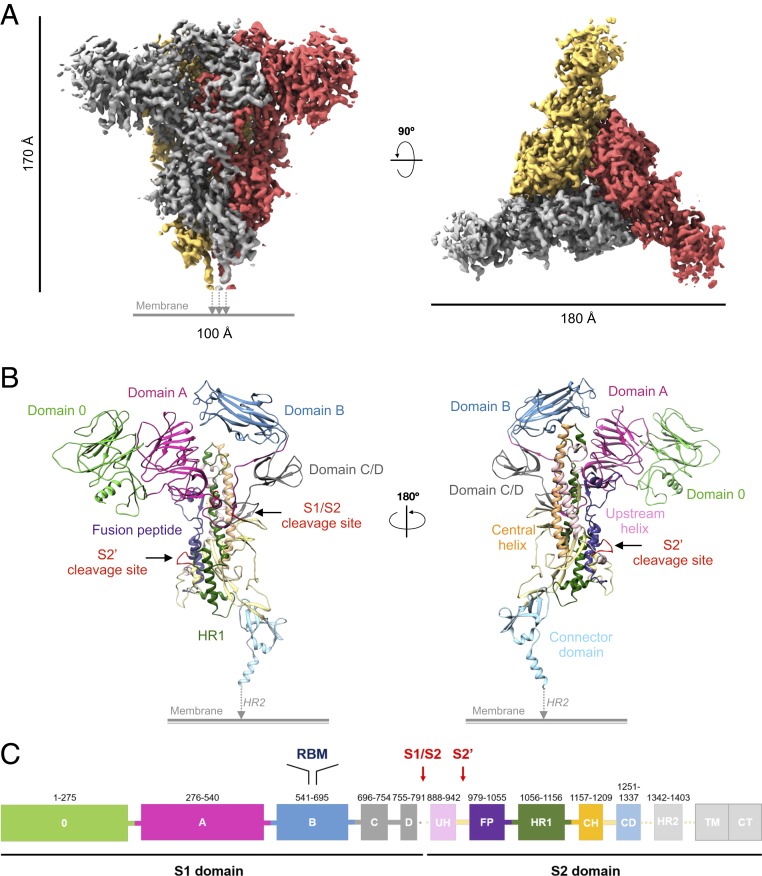
Cryo-EM structure of FIPV-UU4 S protein. (*A*) The 3.3-Å cryo-EM map of FIPV-UU4 S protein shown in side view (*Left*) and top view (*Right*) with the 3 protomers colored in gold, red, and gray. (*B*) Cartoons representative of the atomic model of monomeric FIPV-UU4 S protein. (*C*) Functional subunits and domains are indicated and colored as defined in the schematic representation as a function of the sequence number indicated above each functional unit. CD, connector domain; CH, central helix; CT, cytoplasmic tail; FP, fusion peptide; TM, transmembrane domain; UH, upstream helix. Regions that were resolved by cryo-EM, namely HR2, TM, and CT, are shown in light gray.

The ectodomain of FIPV-UU4 S protein was fused with a trimerization domain of T4 fibritin followed by a V5 tag and a His_6_ tag at the C terminus to replace the transmembrane helix. The recombinant FIPV-UU4 S protein was overexpressed in a human embryonic kidney 293 (HEK 293) cell line. With 1,391 amino acids in its sequence, the expected molecular mass of the homotrimeric recombinant protein is 465 kDa. Nevertheless, size-exclusion chromatography coupled with multiangle static light scattering (SEC-MALS) indicated an apparent molecular mass of 722 kDa for the single elution peak, of which 192 kDa (27% of the total molecular mass) was ascribed to glycan moieties (*SI Appendix*, Fig. S1*A*). The extensive glycosylation and the trimeric state were confirmed by gel electrophoresis under denaturing and native conditions, respectively (*SI Appendix*, Fig. S1*B*). Importantly, immunocytochemistry analysis demonstrated that mouse anti–FIPV-UU4 S protein serum was able to differentiate serotype I- and II-infected cells, thus establishing the potential of FIPV-UU4 S protein as a potent vaccine candidate for FIP treatment (*SI Appendix*, Fig. S1 *C* and *D*). Using a 200-keV electron microscope, we determined the 3D electron density map of the FIPV-UU4 S protein to 3.3-Å resolution, which revealed a propeller-like homotrimer structure with an overall dimension of 180 Å in diameter and 170 Å in height (Fig. 1*A*, Table 1, *SI Appendix*, Figs. S2*A* and S3 *A* and *B*, and Movie S1). In contrast, all previously reported cryo-EM structures of CoV S proteins have adopted compact ellipsoidal shapes of which the host receptor-recognizing S1 subunits pack against the spring-loaded coiled-coil S2 subunits (*SI Appendix*, Table S1) (3, 17–21). Local resolution analysis indicated that the propeller-like regions of the EM map were less defined compared with that of the core regions, indicative of local dynamics (*SI Appendix*, Fig. S3*E*).

**Table 1.** Parameters of cryo-EM data collection, processing, and model validation

Detection mode	Defocus phase contrast	Volta phase plate
Microscope	Talos Arctica	Titan Krios
Voltage, keV	200	300
Defocus range, μm	1.8 to 2.8	0.2 to 0.8
Movies	2,436	1,405
Frames per movie	32	30
Exposure time per frame, s	2.5	2.27
Magnification	120k	120k
Pixel size, Å	0.87	0.85
Total accumulated dose, e^−^/Å^2^	48	30
Particles	102,586	191,810
Map resolution, Å	3.3	3.7
Map-sharpening B factor, Å^2^	−90	−143
3DFSC (sphericity)	0.91	
Cryo-EF (efficiency)	0.66	
Model composition		
Nonhydrogen atoms	32,115	
Protein residues	3,735	
Ligands	219	
B factor, Å		
Protein	170.75	
Ligand	150.62	
Model validation		
MolProbity score	2.12	
EMRinger score	2.62	
CC (mask)	0.79	
d 99 (Å)		
Masked	4.1	
Unmasked	3.9	
d FSC model, 0/0.143/0.5 Å		
Masked	3.1/3.4/3.9	
Unmasked	3.2/3.5/4.2	
Ramachandran, %		
Favored	87.33	
Allowed	12.43	
Outliers	0.24	
Rotamer outliers, %	0.82	
Clashscore	9.50	
Rms deviations		
Bond length, Å	0.011	
Bond angle, °	1.604	

The high-resolution EM map enabled ab initio model building and refinement to generate an atomic model of FIPV-UU4 S protein. The 3 monomers are intertwined through extensive interactions between the conserved S2 subunits, while the 3 S1 subunits form the propeller blades (Fig. 1*B* and Table 1). Detailed inspection of the EM map showed well-defined side-chain densities in most regions, including the interstrand disulfide bonds, attesting to the high quality of our EM-derived structural model (*SI Appendix*, Fig. S3*D* and Movie S2). After considering the electron densities of individual amino acids, many electron densities remained unaccounted for. It transpired that these additional densities correspond to the N-glycan structures (vide infra). Based on our cryo-EM structure, we defined 5 distinct domains within the S1 subunit: domain 0 (residues 1 to 275), domain A (residues 276 to 540), domain B (residues 541 to 695), domain C (residues 696 to 754), and domain D (residues 755 to 791). The S2 subunit consists of an S2′ cleavage site (residues 969 to 978), a fusion peptide (residues 979 to 1055), and 2 heptad repeats (HR1 and HR2), corresponding to residues 1056 to 1156 and 1342 to 1403, respectively. The electron density map corresponding to the HR2 domain of the C terminus (residues 1338 to 1391) could not be resolved, suggesting conformational heterogeneity that leads to loss of contrast after averaging over a large number of particle images (Fig. 1*C*). Likewise, the model of the 4 loop segments (residues 1 to 61, 391 to 410, 781 to 796, and 1337 to 1361) could not be built because of the loss of the corresponding EM maps, potentially due to intrinsic dynamics.

### Site-Specific N-Glycosylation Mapped by MS and Cryo-EM.

**Fig. 2.**
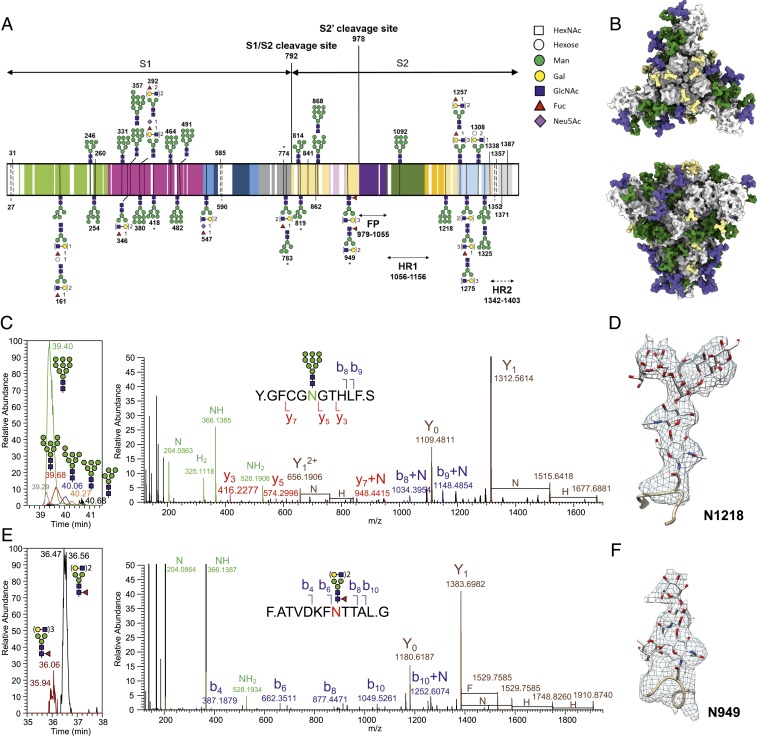
Structural analysis of N-glycosylation of FIPV-UU4 S protein by mass spectrometry and cryo-EM. (*A*) Site-specific annotation of glycan structures. The positions of individual N-glycosylation sites are indicated by their residue numbers along the primary sequence of FIPV-UU4 S protein, which is colored using the same scheme as in Fig. 1*D*. Glycan structures that were confirmed by glycopeptide analysis with high confidence are shown schematically with the individual symbols defined (*Right*). Some glycosylation sites exhibit heterogeneous glycan structures, which are stacked onto each other at the same site. Protein sequences that were not covered by tryptic digests are shown in white. Positions that are predicted to be N-glycosylated but were not experimentally verified are indicated by dashed lines. (*B*) Spatial distributions of experimentally identified glycosylation patterns on FIPV-UU4 S protein shown in 2 orthogonal views as a homotrimer. Mass spectrometry-identified high-mannose and complex/mixed-type glycans are colored in green and magenta, respectively. N-glycosylation that was observed by cryo-EM but not by mass spectrometry are shown in basic type and colored in pale yellow. GlyProt was used to generate the most common atomic structures of high-mannose and complex-type glycans in order to illustrate the extent of the most probable glycan distributions over the surface of the FIPV-UU4 S protein. (*C* and *E*) Overlaid extracted ion chromatograms (*Left*) for the major glycoforms identified on N1218 (*C*) and N949 (*E*) are shown along with the HCD MS/MS spectra of the doubly charged glycopeptides (*Right*), corresponding to the most abundant glycoforms. The accurately measured *m/z* of peptide b and y ions with and without carrying the single HexNAc at the Asn, together with the Y_0_ (peptide backbone) and Y_1_ (peptide backbone + HexNAc), allowed unambiguous assignment of the nontryptic glycopeptides. The glycan compositions were inferred from molecular masses alone and annotated using the standard Symbol Nomenclature for Glycans as high-mannose (Man_9_GlcNAc_2_) and core-fucosylated biantennary complex-type N-glycans, respectively. Annotation of the fragment ions: F, fucose; H, hexose; N, *N*-acetylhexosamine (HexNAc). (*D* and *F*) Expanded views of cryo-EM maps corresponding to the high-mannose structure on N1218 (*D*) and complex-type structure on N949 (*F*). Note that a clear protrusion next to the stem HexNAc was observed, which was assigned to be core fucosylation, in line with the mass spectrometry data (*E*). Both *D* and *F* were derived from the 3.3-Å map (DPC dataset).

Glycosylation of CoV S proteins is implicated in protein folding (22), structure stability (22), virus entry, and receptor recognition (23–27). According to the sequence, 37 N-linked glycosylation sites are predicted for FIPV-UU4 S protein. Liquid chromatography-tandem mass spectrometry (LC-MS/MS) analysis of the de–N-glycosylated tryptic peptides led to identification of 29 occupied N-glycosites based on the conversion of Asn to Asp upon PNGase F treatment (Fig. 2*A*). The remaining 8 predicted N-glycosylation sites, namely ^27^NTSH, ^31^NNSK, ^482^NYTD, ^585^NGSV, and ^590^NVTS in the S1 subunit, ^1308^NTTH in the S2 subunit, and ^1352^NQTK and ^1357^NLTA in HR2, were not verified due to the lack of corresponding tryptic peptides. To obtain an unbiased view of the range of N-glycans carried, the pool of released N-glycans was profiled by MALDI-MS after permethylation. Both high-mannose and ±core-fucosylated, ±sialylated complex-type N-glycans were evidently present, with Man_9_GlcNAc_2_ (M9) and a simple core-fucosylated biantennary structure contributing to the 2 most intense molecular ion signals detected (*SI Appendix*, Fig. S4). Taking the average size of these 2 predominant N-glycan structures—1,768 Da for biantennary and 1,864 Da for M9—that were released from the FIPV-UU4 S protein, a total of 37 glycosylated sites would contribute to a total of 66.6 kDa of glycan per monomer, namely ca. 200 kDa for a trimer, consistent with the 192-kDa glycan contribution as inferred from SEC-MALS data (*SI Appendix*, Fig. S1*A*). Collectively, it confirms that FIPV-UU4 S protein is indeed heavily N-glycosylated at most, if not all, of the predicted sites.

The extensive N-glycosylation of FIPV-UU4 S protein was evident in the 2-dimensional classifications of the raw cryo-EM particle images. The use of the Volta phase plate (VPP) in combination with a 300-keV electron microscope enhanced the contrast of the blurry density around the core protein densities. The VPP-derived dataset was used to construct a 3D EM map, which showed better-defined protrusions with lower local resolutions as a result of conformational heterogeneity (*SI Appendix*, Figs. S2*B* and S5). Through different image-processing procedures, we could unambiguously build 28 N-linked glycan structures onto the atomic model, including 2 N-glycosylation sites, N585 and N590, which were not identified by LC-MS/MS analysis of the de–N-glycosylated peptides (*SI Appendix*, Fig. S6). The reconstituted glycan EM densities were mostly limited to 1 or 2 *N*-acetylglucosamine (GlcNAc) moieties linked to the asparagine side chains due to their intrinsic dynamics. Nonetheless, a number of well-defined glycan densities could be traced and modeled with up to 7 monosaccharide residues. In the case of N357 and N1218, we could resolve the densities that correspond to 2 GlcNAcs and up to 5 mannoses, which is indicative of these 2 sites being mostly glycosylated by high-mannose-type structures (Fig. 2 *D* and *F* and Movie S3).

To further define the distribution of high-mannose versus complex-type N-glycans over the various sites, tryptic digests of FIPV-UU4 S protein were subjected to LC-MS/MS analysis without first removal of the N-glycans. By directly identifying the intact glycopeptides, the site-specific N-glycosylation pattern of 24 sites could be profiled, including ^482^NYTD and ^1308^NTTH, not detected by previous analysis of de–N-glycosylated peptides. This brings the total of MS-verified N-glycosylation to 31 out of the predicted 37 sites (summarized in Fig. 2*A*). For each of the identified sites, the most abundant and hence most representative glycoforms were deduced from the relative peak intensities of the manually extracted ion chromatograms (XICs) of the corresponding glycopeptides (*SI Appendix*, Table S2). For example, the XICs of glycopeptides derived from N1218 and N949 indicated that the major glycoforms correspond to an M9 high mannose and a nonsialylated, fucosylated biantennary complex-type glycan structure, respectively (Fig. 2 *C* and *E*, *Left*). Close examination of the EM density protruding from the side chain of N949 revealed additional density next to the first GlcNAc moiety linked to N949. We attributed this to a core fucose (Fuc), which is consistent with and fully corroborated by the corresponding glycopeptide MS/MS spectrum, which contained a diagnostic peptide backbone fragment ion carrying a GlcNAc and a Fuc (Y_1_+F; Fig. 2*E*).

**Fig. 3.**
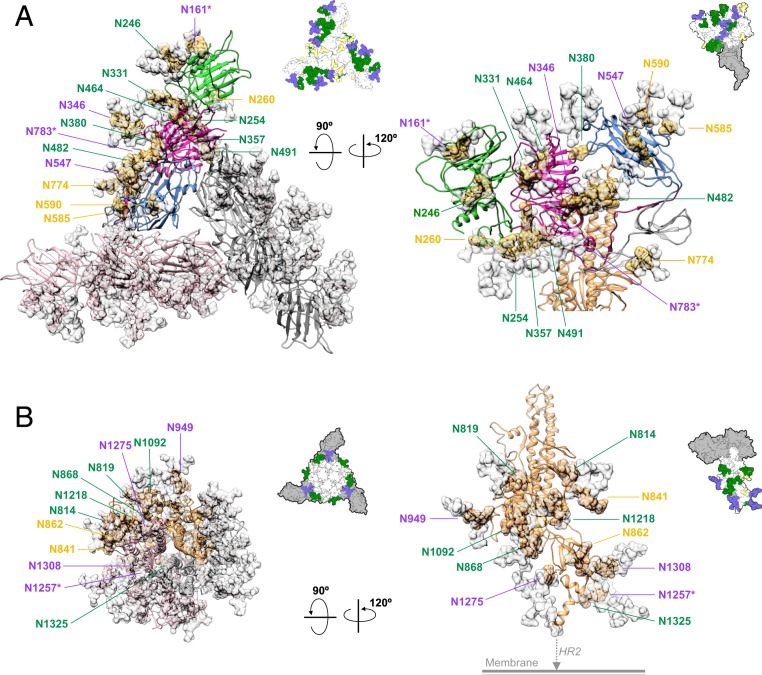
Structural mapping of N-glycosylation on S1 and S2 subunits. Cartoon representations of the protein parts of S1 (*A*) and S2 (*B*) subunits with surface rendering of cryo-EM–observed (gold, salmon, and gray in each of the 3 protomers) and expected (MS-verified and modeled by GlyProt; semitransparent white) N-glycans. For the S1 subunit, 1 protomer is colored in accordance with Fig. 1*B*. The identities of individual N-glycosylated residues within 1 protomer are indicated in green, magenta, and orange, corresponding to high-mannose, complex or mixed, and those that were only observed by cryo-EM, respectively. To guide visualization, the same views of the overall glycosylation model are shown (*Upper Right*) with the same coloring scheme as in Fig. 2*B*. Regions that are not shown in *A* or *B* are shaded gray.

Combining all our cryo-EM and MS data, 33 out of the 37 predicted N-linked glycosylated sites were identified, including 18 and 15 sites in the S1 and S2 subunits, respectively (*SI Appendix*, Table S3). The 4 unaccounted sites included N27 and N31 at the N terminus and N1352 and N1357 at the C terminus, all of which were not resolved by cryo-EM. Based on the chemical structures of the most populated N-glycans derived from glycopeptide analysis and the complementary cryo-EM results, we generated an atomic model that represented the most likely spatial distribution of the N-glycans on the FIPV-UU4 S protein (Figs. 2*B* and 3 and *SI Appendix*, Table S3). These glycans shield more than 2/3 of the protein surface, potentially camouflaging protein functional motifs to evade detection by the host immune system.

### Structural Characteristics of Domain 0 Unique to Alphacoronaviruses.

**Fig. 4.**
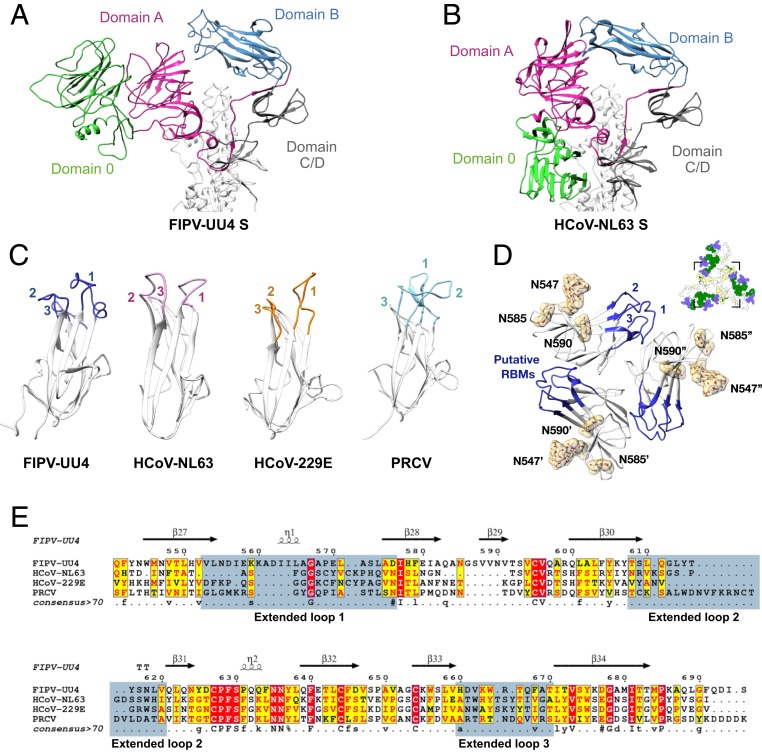
Structure divergence of host receptor recognition motifs among different CoVs. (*A* and *B*) Cartoon representations of FIPV-UU4 (*A*) and HCoV-NL63 (*B*) S1 subunits. The individual domains are colored in accordance with Fig. 1*B*. Note that domain 0 exhibits major conformational rearrangements between FIPV-UU4 and HCoV-NL63. (*C*) Structural comparison of domain B of FIPV-UU4, HCoV-NL63 (PDB ID code 3KBH), HCoV-229E (PDB ID code 6ATK), and PRCV (PDB ID code 4F5C). Extended loops 1 to 3 that are putative host receptor recognition motifs are highlighted with different colors and indicated with the respective numbers. (*D*) Expanded top view of the putative receptor-binding motifs of FIPV-UU4. The only 3 N-glycans in domain B are located on N547, N585, and N590, all of which are distant from the putative RBMs. A schematic view of the overall structure (the same as in Fig. 2*B*) indicates the absence of N-glycans in the putative RBMs. All N-glycan densities were segmented from the 3.3-Å map. (*E*) Sequence alignment of the domain B regions of FIPV-UU4 (residues 541 to 695), HCoV-NL63 (residues 481 to 616), HCoV-229E (residues 290 to 435), and PRCV (residues 283 to 430). Regions that correspond to the 3 extended loops are highlighted in blue.

Compared with the cryo-EM structure of the S protein of human CoV NL63 (HCoV-NL63), which represents the only reported alphacoronavirus S-protein structure (19), domain 0 of FIPV-UU4 is rotated 90° with respect to the adjacent domain A (Fig. 4 *A* and *B*). Such a pronounced conformational difference could stem from the extensive glycosylation at N254, N357, and N491 that wedge between domains 0 and A (Fig. 3*A*). Domain 0 is only present in alphacoronaviruses, while it is structurally similar to domain A of other CoV genera (*SI Appendix*, Table S4). Structure-based bioinformatics analysis indicated that both domains 0 and A of FIPV-UU4 S protein are structurally homologous to galectins, and galectin-4 in particular (*SI Appendix*, Tables S4 and S5). Nevertheless, domains 0 and A of FIPV-UU4 S protein have distinct glycosylation patterns, leading to differential steric hindrance and physiochemical characteristics on their surfaces that may influence host recognition (Figs. 2*A* and 3*A* and *SI Appendix*, Tables S2 and S3).

### Glycan Array Analysis of Domain 0 Unique to Alphacoronaviruses.

Despite sharing a similar 3D fold with galectins (*SI Appendix*, Table S4), domain 0 of FIPV-UU4 S protein lacks the conserved tryptophan residues that galectins use to bind β-galactoside through CH–π interactions (28). Furthermore, the putative carbohydrate-binding pocket in domain 0 is sequestered by a long loop in the cryo-EM structure. Nonetheless, the ill-defined electron density was indicative of local dynamics such that the putative carbohydrate-binding pocket may still be accessible in solution (*SI Appendix*, Table S4).

Because hemagglutinin activity has been reported for several CoVs, we asked whether individual domains of FIPV-UU4 S protein indeed exhibit lectin activity as a way to bind to host cell-surface glycans for mediating FIPV infection. We employed an array of 100 glycan structures to test the lectin activities of full-length FIPV-UU4 S protein and 3 truncated variants, namely domain 0 only, domains 0 and A, and domains 0, A, and B (*SI Appendix*, *Materials and Methods*). Three groups of glycan structures were found to be recognized by all 4 variants, namely sialylated or core-Fuc Galβ(1→4)Glcβ-core structures, sialylated Galβ(1→3)GalNAcβ-core structures, and oligo-glucose (Glc) structures (*SI Appendix*, Fig. S7). Notably, NeuAc sialylation at the 6 position of the inner GalNAc seems to be required (compound 67) while additional sialylation at the 6 position of Gal (compound 69) increased its affinity slightly. However, sialylation at the 6 position of Gal itself without the presence of sialylation on GalNAc (compound 68) did not support binding, whereas the additional presence of sialylation at the 3 position of Gal (compound 70) actually inhibited the binding slightly. Collectively, the glycan array data suggest that positive recognition and binding by domain 0 prefer a minimum Galβ(1→3)GalNAcβ-core structure sialylated at the 6 position of GalNAc. Di- and tri-Glc with α-linkages could be recognized. The structural basis and functional implications of recognizing these sialylated disaccharides in the context of FIPV host recognition remain to be established.

### Structure Comparison of the Putative Receptor-Binding Domain B.

Previous studies on CoVs indicated that domain B within subunit S1 is responsible for primary host receptor binding (1). The structures of domain B from different CoVs share the same overall β-sandwich fold structure, encompassing 2 3-stranded antiparallel β-sheets with divergent loop sequences and conformations that constitute the putative receptor-binding motifs (Fig. 4*C*). The RBMs play a key role in host specificity; mutations in them are responsible for interspecies transmission (29). Among the 4 selected alphacoronaviruses, FIPV-UU4, HCoV-NL63, human CoV 229E (HCoV-229E), and porcine respiratory coronavirus (PRCV), FIPV-UU4 S protein has the longest loop 1 in domain B with a distinct helical conformation (Fig. 4 *C*–*E* and *SI Appendix*, Table S6), rendering it more solvent-exposed and potentially more accessible to host receptors than that of HCoV-NL63 (19). Meanwhile, other regions of domain B of FIPV-UU4 S protein are shielded by N-glycans (Fig. 4*D*).

### Structural Characteristics of the S2 Subunit in Its Prefusion State.

CoVs share conserved sequences and structures of their S2 subunits (30, 31). The S2 subunit of FIPV-UU4 is structurally similar to that of HCoV-NL63 and porcine deltacoronavirus (PdCoV), with which FIPV-UU4 shares 57 and 52% sequence identity, respectively. FIPV-UU4 S protein shows a positional rmsd of 3.3 and 2.2 Å with respect to that of HCoV-NL63 and PdCoV, respectively (*SI Appendix*, Table S7). Our cryo-EM analysis identified 12 N-glycosylation sites in the S2 subunit of FIPV-UU4. Except for N841 and N862, all these N-glycosylation sites were confirmed by MS, including 6 high-mannose types, 3 complex types, and 1 that showed a mixture of high-mannose and complex-type N-glycans (Fig. 3*B* and *SI Appendix*, Tables S2 and S3).

**Fig. 5.**
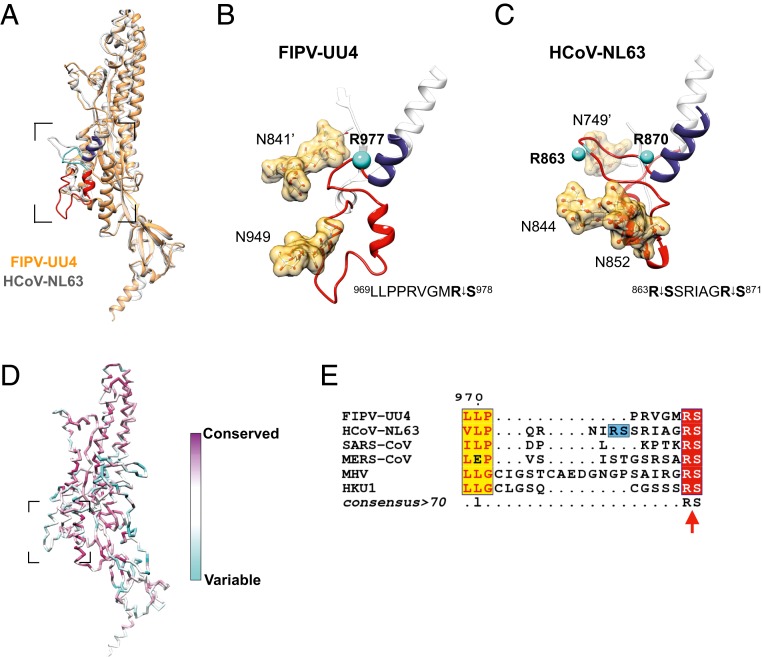
Structural divergence within the S2 subunit of FIPV-UU4 and HCoV-NL63. (*A*) Superimposition of monomeric S2 subunits of FIPV-UU4 (orange) and HCoV-NL63 (semitransparent gray) highlighting the overall structural similarity between the two. The S2′ activation loop, S2′ cleavage sites, and parts of the fusion peptide are colored red, cyan, and indigo, respectively. (*B* and *C*) Expanded views of the CoV activation motifs of FIPV-UU4 (*B*) and HCoV-NL63 (*C*). The respective activation peptide sequences are shown (*Lower Right*) with the protease-specific digestion site shown in bold font indicating the positions of the peptide bond that connects the arginine and serine residues. The Cα-atoms of the corresponding arginine residues are shown as cyan spheres in the 3D model with their identities indicated. The cryo-EM–observed electron densities of the N-glycans are shown in gold surfaces. N-glycan densities of FIPV-UU4 were segmented from the 3.3-Å map. (*D*) Structural mapping of the conservation score of the coronavirus S2 subunit analyzed by the ConSurf server. (*E*) Sequence alignment of the coronavirus S2′ activation loop was generated by ESPript 3.0. The red arrow indicates the S2′ cleavage site. An additional S2′ cleavage site (^863^R^↓^SSR) of HCoV-NL63 is highlighted in blue in the background.

Activation of CoV S proteins through site-specific proteolysis at the conserved S1/S2 and S2′ cleavage sites is an essential step for viral entry. The S2′ cleavage site of FIPV-UU4 (^969^LLPPRVGMR^↓978^S), which connects the upstream S2′ activation loop and a fusion peptide, is implicated in the pathogenicity of serotype I FIPVs (2). The underscore refers to the recognition residue for protease cleavage. Compared with other CoVs, FIPV-UU4 has a short S2′ activation loop with only 1 N-glycan (N949), located upstream of the cleavage site (R977; Fig. 5). Another N-glycan stemming from the other monomer (N841′) is in close proximity to the S2′ cleavage site. These 2 N-glycans may provide steric hindrance for proteolysis of the S2′ activation loop, thereby changing the protease activation requirements and host cell tropism (2).

### Bioactivity of FIPV-UU4 S Protein.

To investigate the antigenicity of the HEK293-expressed recombinant FIPV-UU4 S protein, the recombinant FIPV-UU4 S protein was used to immunize mice. The mouse serum was collected and subjected to immunohistochemistry (IHC) staining of clinical serotype I FIPV-infected cat tissues, which were characterized by typical granulomatous inflammation, and the immunocytochemistry (ICC) staining of serotype II FIPV (NTU156)-inoculated Fcwf-4 cells. The result showed positive cytoplasmic signals in macrophages associated with granulomatous lesions by IHC staining but negative signals in serotype II FIPV NTU156-inoculated Fcwf-4 cells by ICC staining, confirming that the recombinant FIPV-UU4 S protein indeed exhibits antigenicity that is close to that of native serotype I FIPVs (*SI Appendix*, Fig. S1 *D* and *E*).

## Discussion

We report here a high-resolution cryo-EM structure of FIPV-UU4 S protein with a distinct domain architecture that sets it apart from all previously reported CoV S protein structures (Fig. 1 and *SI Appendix*, Table S2). The most significant structural difference is the unique 3-blade propeller-like structure formed by the S1 subunit. The combined efforts of cryo-EM and MS analyses identified and delineated the positions and structures of 33 out of the 37 predicted N-glycosylation sites, accounting for 27% of the total molecular mass. Our results represent the unprecedentedly detailed analysis of the N-linked glycans of a CoV S protein in terms of their chemical compositions and 3D structures, made possible through the use of a mammalian expression system to introduce physiological mammalian glycosylation patterns. The resulting models revealed some spatial partitioning of high-mannose versus complex-type glycans. Several high-mannose-type glycans are located at the junctions between domains and appear to serve as wedges to maintain the domain architecture (Fig. 2*B*). It is plausible that the protein domain junctions generate significant steric hindrance, thereby preventing subsequent trimming and addition of the complex-type glycan structures (32).

In addition to protein folding, viral protein glycosylation plays a pivotal role in viral infection. Glycosylation of virus envelope proteins helps shield their antibody-neutralizing epitopes as a way to evade host immunity. One of the best-known examples is the envelope glycoprotein (gp120) of HIV-1, of which glycosylation accounts for almost half of its molecular mass and plays an essential role in host immunity evasion (33). In CoVs, a recent study on HCoV-NL63 suggests that the glycosylation at N358 within its RBMs may serve as molecular trickery for evading host immunity (19). However, the role of the absent glycan shield at the corresponding putative RBMs in FIPV-UU4 S protein should be further investigated.

In addition to their protective roles in shielding viruses from being neutralized by host immune systems, spike-protein glycosylation can be selectively recognized by host cells to facilitate viral infections. Lectin-dependent enhancements of viral infections have been reported for SARS-CoV (34), Ebola virus (35, 36), Marburg virus (34), and Dengue virus (37). Both serotype I and serotype II FIPVs use feline dendritic cell-specific intercellular adhesion molecule 3-grabbing nonintegrin (fDC-SIGN) as a coreceptor to recognize high-mannose glycans during viral entry and transmission (34). Domain A of FIPV-UU4 S protein is densely decorated with high-mannose-type glycans, which could be implicated in interacting with fDC-SIGN during viral infection (Figs. 2*A* and 3*A*).

While viral envelope or S-protein glycosylation is targeted by host cells, several viral envelope or S proteins also exhibit lectin activities to recognize host surface glycans in trans (38). For example, a number of CoVs have been reported to exhibit hemagglutinin activity with some preference for sialylated oligosaccharides (39). Through glycan array analysis, we obtained experimental evidence of lectin activity for domain 0 of FIPV-UU4 S protein, which showed a distinct binding preference for a Galβ(1→3)GalNAcβ-core structure sialylated at the 6 position of the inner GalNAc. If one disregards the anomericity of the GalNAc, this minimal NeuAcα(2→6)GalNAcα-determinant corresponds to the sialyl Tn epitope widely implicated as a cancer and CART antigen, and also being developed as a vaccine candidate. Follow-up binding studies including the use of custom-made O-glycans containing the actual sialyl Tn epitope and other core 1 O-glycans sialylated at different positions would be required to substantiate this intriguing finding that sialylated O-glycans on host cell surfaces might play an important role in viral recognition and infection of serotype I FIPV.

In the present study, 33 N-glycosylation sites were confirmed on the ectodomain of the trimeric S protein. M9 high mannoses were identified on N1092 and N1218 (*SI Appendix*, Fig. S8) of the Th1 and/or Th2 epitopes (residues 1051 to 1110 and 1208 to 1235) of the FIPV-UU4 S protein (40). Viral protein glycosylation might determine the host tropism (41), immunogenicity, and pathogenicity (42–46). The N-linked glycans in Th1 epitopes might function as hindering structures that constrict T cell recognition or mislead the host immune defense by producing an ineffective immune response (42–46), as demonstrated in HIV (47). The role of these glycans in the pathogenesis of FIP should be further studied.

In summary, we describe, at the atomic level, the structure and glycosylation of FIPV-UU4 S protein. This represents structural work on the serotype I FIPV family, which inflicts an exceptionally high mortality rate for infected cats. The structure reveals major structural differences in the S1 subunit from the only known example within the alphacoronavirus genera, HCoV-NL63 S protein, for which structural information is available. These results demonstrate how structurally divergent these S proteins can be despite their sequence homology (*SI Appendix*, Table S1). The integrated use of cryo-EM and MS reveals the unique domain architecture with detailed structural information regarding its glycosylation, the galectin-like N-terminal domains, and the location of the putative RBMs of serotype I FIPV. The high-resolution structure of FIPV-UU4 S protein may therefore serve as the blueprint for further mechanistic insights into viral host interactions of FIPV.

## Materials and Methods

### Purification of FIPV-UU4 S Protein.

HEK293 cells stably expressing FIPV-UU4 S protein were washed by Dulbecco’s phosphate-buffered saline (PBS) (Gibco) 2 times prior to inoculation into 9 175T flasks, each containing 350 mL FreeStyle 293 expression medium (Gibco), and cultured in the CELLSPIN System (INTEGRA Biosciences) at 37 °C for 5 d. Cells were harvested by centrifugation at 1,000 rpm for 20 min, and the supernatant was collected and filtered through a membrane with a pore size of 0.22 μm. The supernatant was supplemented with 10× binding buffer (500 mM sodium phosphate, 1.5 M sodium chloride, 100 mM imidazole, pH 8). HisPur cobalt resin (Thermo Fisher Scientific) was added to the supernatant (20 mL resin per L) following the manufacturer’s protocols. The eluent was concentrated using Vivaspin 20 (GE Healthcare) with 100-kDa molecular mass cutoff, supplemented with cOmplete EDTA-free Protease Inhibitor Mixture (Roche), aliquotted, flash-frozen in liquid nitrogen, and stored at −80 °C until further use.

#### Cryo-EM sample preparation and data collection.

##### Defocus phase-contrast cryo-EM.

Four microliters of purified FIPV-UU4 S protein (0.2 mg/mL) in PBS (Sigma-Aldrich; 79382) was applied onto glow-charged 200-mesh Quantifoil R2/1 holey carbon grids. The grids were blotted for 3 s at 4 °C and 100% humidity, and vitrified using a Vitrobot Mark IV (Thermo Fisher). Cryoelectron data of FIPV-UU4 S protein were collected using a 200-keV Talos Arctica microscope with an exposure time of 2.5 s and pixel size of 0.87 Å, using a Falcon III detector (Thermo Fisher) in a linear mode. In total, 2,436 micrographs were collected with defocus ranging between 1.8 and 2.8 µm and accumulated exposure of 48 e^−^/Å^2^ distributed over 32 frames.

##### Volta phase plate cryo-EM.

The grid preparation was the same as aforementioned for defocus phase-contrast (DPC) cryo-EM data collection. Cryoelectron micrographs of FIPV-UU4 S protein were collected using a 300-keV Titan Krios microscope (Thermo Fisher) with an exposure time of 2.27 s and pixel size of 0.85 Å, using a Falcon III detector (Thermo Fisher) in a linear mode with contrast enhancement by using a VPP. In total, 1,405 micrographs were collected with defocus ranging between 0.2 and 0.8 µm and accumulated exposure of 30 e^−^/Å^2^ in 30 output frames.

### Data Availability.

The atomic coordinates of FIPV-UU4 S protein have been deposited in the Protein Data Bank (PDB) under ID code 6JX7. The cryo-EM maps, including unsharpened and sharpened maps, have been deposited in the Electron Microscopy Data Bank (EMDB) under ID code EMD-9891.

Further detailed information on materials and methods is provided in *SI Appendix*, *Materials and Methods*.

## Supplementary Material

Supplementary File

Supplementary File

Supplementary File

Supplementary File
