# Supplementary material for: Cryo-EM analysis of a feline coronavirus spike protein reveals a unique structure and camouflaging glycans
Source: Proc Natl Acad Sci U S A. 2020 Jan 3;117(3):1438–46. doi: 10.1073/pnas.1908898117 (PMC6983407; doi:10.1073/pnas.1908898117)
Supplement: Supplementary File [file pnas.1908898117.sapp.pdf]

PNAS
www.pnas.org

Supplementary Information for

Cryo-EM Analysis of a Feline Coronavirus Spike Protein Reveals a Unique Structure and Camouflaging Glycans

Tzu-Jing Yang[1,2,#] & Yen-Chen Chang[1,3,#], Tzu-Ping Ko[1], Piotr Draczkowski[1], Yu-Chun Chien[1,2], Yuan-Chih Chang,[4] Kuen-Phon Wu[1], Kay-Hooi Khoo[1,2],
Hui-Wen Chang[3,*] & Shang-Te Danny Hsu[1,2,*]

Corresponding authors: Hui-Wen Chang and Shang-Te Danny Hsu
Email: Shang-Te Danny Hsu (sthsu@gate.sinica.edu.tw) or Hui-Wen Chang (huiwenchang@ntu.edu.tw).

This PDF file includes:

Supplementary text
Figs. S1 to S8
Tables S1 to S7

Other supplementary materials for this manuscript include the following:

Movies S1 to S3

www.pnas.org/cgi/doi/10.1073/pnas.1908898117

Supplementary text

SI Materials and Methods

Plasmid construction of FIPV-UU4 S protein. The nucleotide sequence of the FIPV S protein from strain UU4 (FIPV-UU4; Genbank accession No. FJ938054) is fused to a foldon domain of phage T4 fibritin sequence at the 3' end (1, 2); the nucleotide sequence corresponding to N-terminal signal peptide was replaced by a tissue plasminogen activator signal peptide (tPA-SP) sequence to facilitate secretion (2). The open reading frame of the engineered protein sequence was codon-optimized for human cell expression, and synthesized by Genscript Corporation. The synthetic gene was inserted into pcDNA™ 3.1/V5-His TOPO® vector (Invitrogen) between *BamHI* and *NotI* restriction sites to obtain the plasmid of pcDNA™ 3.1-FIPV-UU4 S-V5-His (Fig. S10), and confirmed by nucleotide sequencing (Tri-I Biotech Inc.).

Establishment of a stable cell line expressing FIPV-UU4 S protein. The plasmid pcDNA™ 3.1-FIPV-UU4 S-V5-His was introduced into human embryonic kidney cell 293 (HEK 293, ATCC® CRL-1573™) by transfection using the GenJet Plus In Vitro DNA Transfection Reagent (SignaGen® Laboratories) according to the manufacturer's protocol. Two days post transfection, the culture supernatant was changed into selection medium containing Dulbecco's modified Eagle's medium (DMEM, Gibco) supplemented with 10% fetal bovine serum (FBS, Gibco) and 750 μg/L Geneticin® Selective Antibiotic (G418, Gibco) for establishing a stable FIPV-UU4 S expressing line.

Evaluation of transfection efficacy by immunocytochemistry staining. The transfected cells were seeded on a 96 well plate (Nunc) and cultured at 37 ºC for 16-18 h to reach 80-90% confluence. For cell fixation, the culture medium was removed, and 200 μL of 80% ice-cold acetone was added to fix the cells for 10 min at -20 ºC. After discarding the acetone and air-drying the plate, 100 μL anti-V5 antibody (1000 dilutions in phosphate-buffered saline, PBS; Invitrogen) was added and incubated at RT for 1 h, followed by washing with 200 μL of PBS for six times, and detection with an anti-rabbit/mouse immunoglobulin EnVision™ + DAB system (Dako) according to the manufacturer's protocol. The result was examined by using an inverted microscope. Cells with brown signals in the cytoplasm were interpreted as a positive result.

Western blotting. Cells were lysed using Total Protein Cell Lysis Buffer (AMRESCO) at 4 ºC for 15 min. The cell lysates were mixed with 10X NuPAGE® Reducing Agent and 4X NuPAGE® LDS Sample Buffer (Thermo Fisher Scientific) were heated at 95 ºC for 5 min, and loaded on a 4-12% protein gel for electrophoresis using Mini-PROTEAN® Tetra Cell Systems (Bio-Rad). After transferring the protein to the Immun-Blot ® PVDF Membrane (Bio-Rad),

the membrane was initially blocked Tris-buffered saline with 0.01% (v/v) Tween 20 (TBST) buffer containing 5% skim milk powder, and stained with anti-V5 antibody (5000 dilutions in TBST) (Invitrogen) at RT for 1 h. After three times of TBST washings, it was stained by horseradish peroxidase conjugated goat-anti-mouse IgG (10000 dilutions in blocking buffer) (Jackson ImmunoResearch laboratories) at RT for 1 h, followed by three times TBST washings. The membrane was incubated with Clarity™ Western ECL Blotting Substrates (Bio-Rad) at RT for 3-5 min as per manufacture's protocol and visualized by ChemiDoc™ Imaging Systems (Bio-Rad) via chemiluminescence.

Native polyacrylamide gel electrophoresis. A 10% acrylamide/Bis-acrylamide (Bio-Rad) separating gel with 4% acrylamide/Bis-acrylamide stacking gel was casted following the manufacturer's protocol. FIPV-UU4 S protein was added with 2X sample buffer and heated at 95 °C for 5 min. The electrophoresis was performed at 180 V for 4 h, and stained with Coomassie Brilliant Blue R-250 Staining Solution (Bio-Rad) and detected by ChemiDoc ™ Imaging Systems (Bio-Rad).

Size-exclusion chromatography-coupled with multiangle static light scattering (SEC-MALS) analysis of FIPV-UU4 S protein. The absolute molecular weight of FIPV-UU4 S protein was determined by SEC-MALS as previously described using an FPLC instrument (UPC10, GE Healthcare) coupled with a multi-angle light scattering detector (DAWN, Wyatt Technology) and a refractive index detector (Optilab T-rEC, Wyatt Technology) (3). Bovine serum albumin, which was used as a reference standard, and FIPV-UU4 S protein, were prepared at a concentration of 2 mg/mL in D-PBS (pH 7.4) with 0.02% NaN_3. SEC was carried out using a Bio-SEC 3 liquid chromatography column (Agilent). To deconvolute the contributions of amino acids and carbohydrates to the molecular weights of FIPV-UU4 S protein, the refractive index increments (dn/dc) of protein and protein conjugate (carbohydrates) were defined as 0.185 and 0.140 mL/g, respectively, as inputs for the molecular weight calculations by using the ASTRA 6.0 software (Wyatt Technology). The buffer viscosity (η) was estimated to be 0.8945 cP at 25°C using SEDNTERP.

Image processing and 3D reconstruction

DPC dataset. Relion-3.0-beta (4) and *cis*TEM (5) were used for data processing. All movies were subject to whole-frame alignment with dose-weighting using MOTIONCOR2 (6). The contrast transfer function (CTF) was estimated by using GCTF (7). Output micrographs were filtered and the outliers discarded. Particles positions were automatically identified using templates generated from a subset of 30 micrographs by Laplacian-of-Gaussian (LoG)-based auto-picking algorithm (4).

The initial dataset of 442,004 particles images was cleaned up by means of 2D and 3D

classification using particle images down-sized by a 2×2 binning (a reduced box size of 220 pixel). 103,279 particle images were selected and re-extracted with their full resolution using a 440-pixel box size. 3D refinement and post-processing of the full-size particle images yielded a map of FIPV-UU4 S protein with a resolution of 3.91 Å. The particle images were further refined by Bayesian particle polishing followed by per-particle CTF refinement. All analyses were carried out by using Relion-3.0-beta (4).

The refined set of particle images was used as inputs for one more round of 2D classification using *cis*TEM. An a*b initio* 3D map was generated from the best 2D classes (102,586 particles) to serve as the input for 3D refinement. After the refinement routine within *cis*TEM, an EM map of 3.31 Å resolution was obtained, which was sharpened by using a B-factor of -90 Å^2. The resolution of the map was estimated based on the gold-standard FSC = 0.143. Local resolution analysis was calculated using ResMap (8). The particle orientation distribution was further assessed by cryoEF (9) and 3DFSC (10).

VPP dataset. Relion-3.0-beta (4) and cryoSPARC v2 (11) were used for VPP data processing. All movies were pre-processed following the procedure as described above, except that only six instead of 30 micrographs were used during the initial template search step. All particles were re-extracted and down-sampled with 2 x 2 binning to yield a pixel size of 1.7 Å with a box size of 220 pixels.

The initial dataset of 240,801 particles images was cleaned up by two rounds of 2D classification by using cryoSPARC: the particle images were down-sized by a 2×2 binning (a reduced box size of 220 pixel). These particle images were subsequently used as inputs for Relion-3.0-beta (4). Data conversion between cryoSPARC v2 and Relion-3.0 and was achieved by using the pyem script developed by Daniel Asarnow (https://github.com/asarnow/pyem). An *ab initio* model was built with a C1 symmetry. Two rounds of 3D classification were performed for further particles cleanup. 191,180 particle images from four good 3D classes were selected and re-extracted with their full resolution using a 440-pixel box size. The full-size particle images yielded a FIPV-UU4 S protein map with a 4.2 Å resolution after 3D refinement and post-processing. The particle images were further corrected by per-particle CTF refinement followed by Bayesian particle polishing. The polished particles were used for further 3D refinement, resulting in a 3.7 Å map after post-processing.

Model building and refinement. The atomic model of FIPV-UU4 S protein was constructed using Phenix (12) and Coot (13). First, an overall poly-alanine backbone was created within the EM density by Phenix. Sequence assignment was carried out manually using Coot, aided by manual inspection for aromatic side chains. A homology model was built using Swiss-Model (14) as a template for manual model building. By dividing the homology into individual domains and manually move them through rigid-body movements using Coot, an initial model

was generated to enable subsequent model building that accounted for conformational changes in the loop regions and individual side-chain rotameric states. Finally, a convergent model was eventually generated, energy minimized and refined. *N*-linked glycan models were generated by using the extension module “Glyco” (15) within Coot. Generally, individual glycan moieties corresponding to the structures determined by MS-based glycopeptide analysis were manually built onto asparagine side-chains within the N-X-S/T motifs using the “Linked Monosaccharide Addition (LMA)” function of the Glyco module within Coot; the number of glycan moieties built on each glycosylation site depended on the extent of resolved electron densities protruding from the asparagine side-chain. The glycosidic linkages were created by pre-defined function of LMA. All glycan trees were refined by using the function “Torsion fit & Refine this residue” of Glyco. The geometries of individual *N*-glycan moieties were subsequently validated by Privateer (16, 17). Iterative model refinements were carried out iteratively by using the real-space refinement of Phenix and Coot. The final model was assessed by MolProbity (18) and EMRinger (19).

To generated an atomic model of a fully glycosylated FIPV-UU4 S protein in accordance with the chemical and structural information derived from MS analysis (vide infra), the atomic coordinates of high mannose and basic type were generated by Glyprot (20) using the aforementioned trimeric FIPV-UU4 S protein model. Complex-type *N*-glycans were created by Sweet-II server (21, 22) with the nomenclatures defined by the Glycosciences.DB entries: 269, 1524, 728 and 2712 as the templates for model building (23). The glycan models were subsequently built onto the corresponding asparagine side chains, and manually refined by Coot to avoid steric clashes. In cases when high mannose and complex type were both detected by MS at a given site, a complex type glycan was modeled; in case when no MS information was available but cryo-EM evidence was present to confirm the presence of *N*-glycosylation, i.e., N260, N585, N590, N774, N841 and N862, a basic type glycan (2 NAG + 3 Mannose) was generated for the modeling. DALI server was used to search for structural homologs of individual domains of FIPV-UU4 S protein (24). Structure-based sequence alignments were performed by using ESPript (25). Structural visualization and rendering of structural representations were performed by using UCSF Chimera (26), ChimeraX (27), and Pymol (Schrödinger Inc. U.S.A.).

De-*N*-glycosylation analysis. Two microliters of the FIPV-UU4 S protein (1.8 mg/mL) was unfolded by adding 25 mM ammonium bicarbonate buffer (ABC buffer) supplemented with 2 mM dithioerythritol (Sigma-Aldrich) and 8 M urea (Sigma-Aldrich). The sample was incubated at 37 ºC for 1 h prior to subsequent addition of ten microliters of 25 mM ABC buffer containing 20 mM iodoacetamide (Sigma-Aldrich) followed by incubation in the dark at RT for another 1 h, after which ABC buffer was added to reduce the final urea concentration to 1 M. The protein was digested at 37 ºC overnight by two combinations of proteases (with a

protein to protease ratio of 20-50:1), namely chymotrypsin (Promega) plus trypsin (Promega), and Arg-C (Promega) plus Asp-N (Promega). After proteolysis, *E. coli* PNGase F (Roche) was added to remove *N*-linked glycans at 37 ºC overnight. The reaction was quenched by adding 1 % folic acid (Sigma-Aldrich). The peptide mixture was vacuum-dried by using a miVac DNA concentrator (Genevac), and desalted using a ZipTip® Pipette Tip (Millipore) according to the manufacturer's protocol.

The peptide mixtures were loaded onto an Acquity BEH C18 column (Waters) for nanoLC−nanoESI-tandem MS (MS/MS) analysis using an LTQ Orbitrap Velos hybrid mass spectrometer (Thermo Fisher) equipped with a PicoView nanospray interface (New Objective). The resulting data were generated by Mascot Daemon (Matrix Science) using default settings, and submitted to Mascot (version 2.6, Matrix Science) for identifications. All MS/MS spectra were searched against the NCBI database (November, 2017) with the following search criteria: 10 ppm precursor mass tolerance; 0.02 Da product ion mass tolerance; variable modification of deamidation, carbamidomethylation and oxidation; two missed cleavage and peptide charge of +2, +3 and +4 were allowed. The results were statistically validated with a threshold of greater than 95% probability.

MS-based profiling of release glycans. FIPV-UU4 S protein was digested by trypsin after reduction and alkylation, followed by PNGase F treatment as described in the previous section. The resulting *N*-glycans and de-*N*-glycosylated peptides mixtures were subjected to simultaneous reduction and release of *O*-glycans in 0.05 M sodium hydroxide/1 M sodium borohydride at 45 ºC for 16 h, followed by desalting through Dowex (H^+ form; Bio-Rad) beads packed on a C18 Sep-Pak cartridge (Waters) in 5% (v/v) acetic acid. After drying, the sample was permethylated as described previously (28). For MALDI-MS analysis on the 5800 MALDI-TOF/TOF system (AB SCIEX), the permethylated glycans were mixed with 2,5-dihydroxybenzoic acid (DHB) matrix solution (10 mg/mL in 50% acetonitrile) prior to spotting onto the target plate. The laser energy was set at 5000, and 4000 shots were accumulated for one spectrum. All glycan peaks were manually assigned and annotated.

Glycopeptide analysis by LC-MS/MS. The FIPV-UU4 S protein was digested by trypsin and chymotrypsin using the same conditions as used for De-*N*-glycosylation analysis but without PNGase F treatment (see above). The digested glycopeptides were dissolved in 0.1% (v/v) formic acid (Solvent A) for nanoLC-MS/MS analysis using an Orbitrap Fusion Lumos mass spectrometer (Thermo Fisher) fitted with an EASY-nLC™ 1200 system. The digests were trapped and separated using a PepMap C18 column (Thermo Fisher) with a flow rate of 300 nL/min, and a segmented gradient of 5 to 45% solvent B (80% acetonitrile with 0.1% formic acid) over 75 minutes. A survey scan was acquired from 400 to 1800 *m/z* at a mass resolution of 120,000 with a target value of 2×10^5 ion count. The precursors with charge state 2 to 10

were isolated in a 3 sec top speed duty cycles. The MS/MS analyses were performed by applying an isolation window of 2 Th in the quadrupole, an HCD fragmentation with 28% normalized collision energy, and mass measured in the Orbitrap at a resolution of 30,000. The MS^2 ion count target value was set to 5×10^4 and the maximum injection time was 65 ms. Dynamic exclusion duration was set to 35 s with a 15 ppm tolerance around the selected precursor and its isotopes.

The HCD MS/MS data were processed by the Byonic software (Protein Metrics Inc.) using the following parameters: search against the FIPV-UU4 S protein sequence with fully specific cleavages at F, Y, W, L, K, R residues, allowing up to 2 missed cleavages, with the precursor ion mass tolerance set at 10 ppm and the fragment ion mass tolerance at 20 ppm. Fixed modification considered was cysteine carbamidomethylation (+57.0215 Da), whereas methionine oxidation (+15.9949 Da) was set as a common modification. The built-in *N*-glycan libraries of "182 human no multiple fucose" or "309 mammalian no sodium" were used. The criteria used in additional manual filtering of positive matches were score >200 and PEP2D <0.001. The accepted peptide-spectrum matches were then manually examined and the ion chromatograms of their respective precursors were extracted at 5 ppm to record the peak intensity of each unique glycopeptide identified.

Immunization of FIPV-UU4 S protein in mice. Two three-month-old female BALB/c mice were first primed by a single intramuscular (IM) injection of 60 µg purified FIPV-UU4 S protein with 100 µL Freund's complete adjuvant (Sigma-Aldrich) following by two boosts of 60 µg purified FIPV-UU4 S protein with 100 µL Freund's incomplete adjuvant (Sigma-Aldrich) by intramuscular and intraperitoneal administrations, respectively. Two weeks after the second boost, serum was collected for determining the antigenicity of FIPV-UU4 S protein in mice. The animal protocol was reviewed and approved by the Institutional Animal Care and Use Committee of National Taiwan University (Taiwan, Republic of China; NTU-103-EL-60).

Immunohistochemistry staining of serotype I FIPV-infected tissue using FIPV-UU4 S protein immunized mouse serum. Representative tissue blocks from two cases of clinically and pathologically confirmed serotype I FIPV infected cats were sliced at 4 µm in thickness, put on silane coated slide (Muto Pure Chemicals). After deparaffinizing the tissue slide by xylene and rehydrating with gradient ethanol, the immunohistochemistry staining was performed as previously described (29) and the primary antibody was replaced by either the FIPV-UU4 S protein immunized mouse serum (1:1000 dilution) or a mouse anti feline coronavirus antibody (clone FIPV3-70, Bio-Rad) (1:200 dilution). The result was determined by light microscope and the positive signal was interpreted by showing brown pigments in the cytoplasm of tissue macrophages.

Detection of cross-reactivity of the FIPV-UU4 S protein immunized mouse serum with serotype II FIPV infected Fcwf-4 cells. To detect the cross-reactivity of the serum derived from FIPV-UU4 S protein immunized mouse with serotype II FIPV, the immunocytochemistry staining of the serum with a serotype II Taiwan NTU156 strain (30) (kindly provided by Prof. Chueh at National Taiwan University) infected Fcwf-4 cells (American Type Culture Collection No. CRL-2787™) was performed. The cells were incubated with 100 μL mouse anti-FIPV-UU4 S serum (1000 dilution in PBS) or the mouse anti feline coronavirus antibody (clone FIPV3-70, Bio-Rad) (1:200 dilution) at RT for 1 h. For developing, the anti-rabbit/mouse immunoglobulin EnVision™ + DAB system (Dako) was used following the manufacture's protocol. The results were evaluated using an inverted microscope. The positive signals were characterized by brown pigments located in the cytoplasm of the syncytium.

Identification of glycan binding ability and location of the FIPV-UU4 S protein. To elucidate the glycan binding ability of the FIPV-UU4 S protein, three variants of truncated FIPV-UU4 S proteins were constructed and purified for glycan array analysis together with full-length protein. Briefly, the oligonucleotide sequences corresponding to the protein sequences of Domain 0 (residues 1-275), Domains 0 and A (residues 1-540), and Domains 0, A and B (resides 1-695) were obtained by using one forward primer (Domain 0 forward primer 5'-AAAACCGAGCTCGGATCCAGTACCCTTC-3') in combination with three different reverse primers (Domain 0 reverse primer 5'-AAAAGCGGCCGCCTGCACAGTAATCAGTATG-3', Domains 0 and A reverse primer 5'-AAAAGCGGCCGCGTGGCATTGTC-3' and Domains 0, A and B 5'-AAAAGCGGCCGCGGCTGATGTCCTG-3') through standard PCR reactions. The resulting oligonucleotides were inserted into the pcDNA™ 3.1 plasmid. The constructs were transfected into FreeStyle 293-F Cells (Thermo Fisher) using 293fectin™ Transfection Reagent following the manufacturer's protocol. The transfected cells were incubated at 37 ºC in 8% CO_2 for five days. The supernatant was harvested after centrifugation of 1000 rpm for 20 min. The recombinant proteins were purified using the same procedure as used for full-length FIPV-UU4 S. The final protein concentrations were adjusted to 0.5 mg/mL. The glycan binding of FIPV-UU4 S variants were assessed by Glycan Array 100 (RayBiotech; https://www.raybiotech.com/glycan-array-100/) and GenePix 4000B Biochip Reader (Molecular Devices) (31, 32).

SI Figures

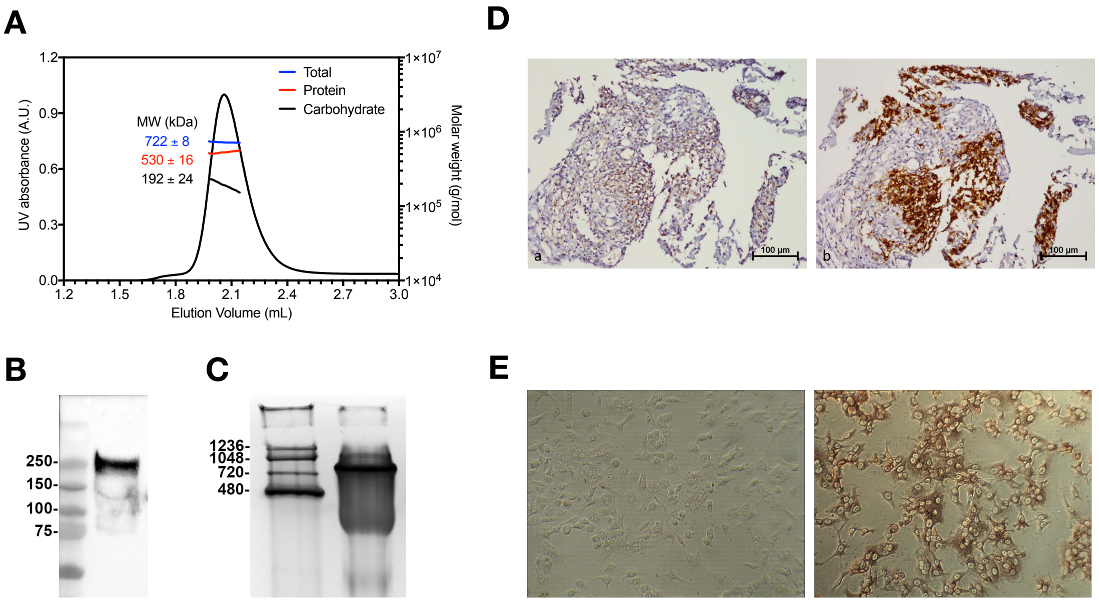

Fig. S1. Biophysical and biochemical characterizations of FIPV-UU4 S protein. (*A*) SEC-MALS analysis reports on the molar mass (g/mol) distributions of total molecule (blue), protein (red) and carbohydrate moiety (black) as a function of elution volume. Carbohydrate moiety accounts for 27% of total MW (192 out of 722 kDa). (*B*) Western blot analysis of the recombinant FIPV-UU4 S protein detected by an anti-V5 antibody. Black arrow indicated the position of monomeric S protein. (*C*) Native gel electrophoresis of the recombinant FIPV-UU4 S protein. Black arrow indicates the position of the band that has a MW higher than 720 kDa, which is interpreted as trimeric FIPV-UU4 S protein. (*D*) Immunohistochemistry (IHC) staining in the natively FIPV-infected feline tissue under 200X magnification. Left and right panels show staining results with 1000X mouse anti-FIPV-UU4 S protein serum and 200X commercial mouse anti-feline coronavirus (anti-FCoV) antibody (positive control), respectively. The intracytoplasmic brown pigments of the macrophages are positive results. (*E*) Immunocytochemistry (ICC) staining of the FECV-infected Fcwf-4 cell. FECV-infected cells stained with 1000X moue anti-UU4 serum and 200X commercial mouse anti-FCoV antibody are shown on the left and right panels, respectively. The brown pigments in the cytoplasm of the syncytial cells are positive signals.

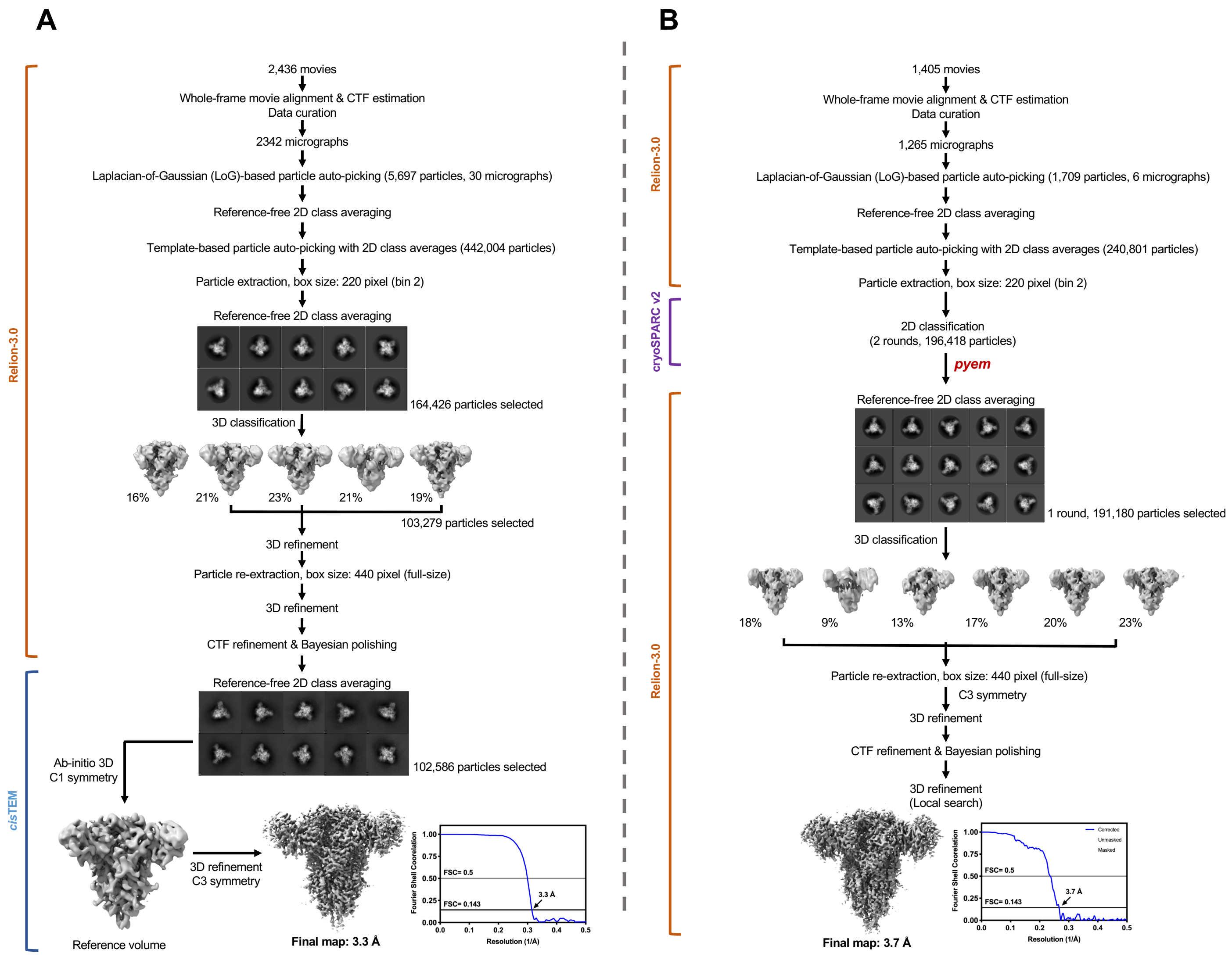

Fig. S2. Cryo-EM data processing workflow for (*A*) regular defocus phase contrast (DPC) dataset and (*B*) Volta phase plate (VPP) dataset.

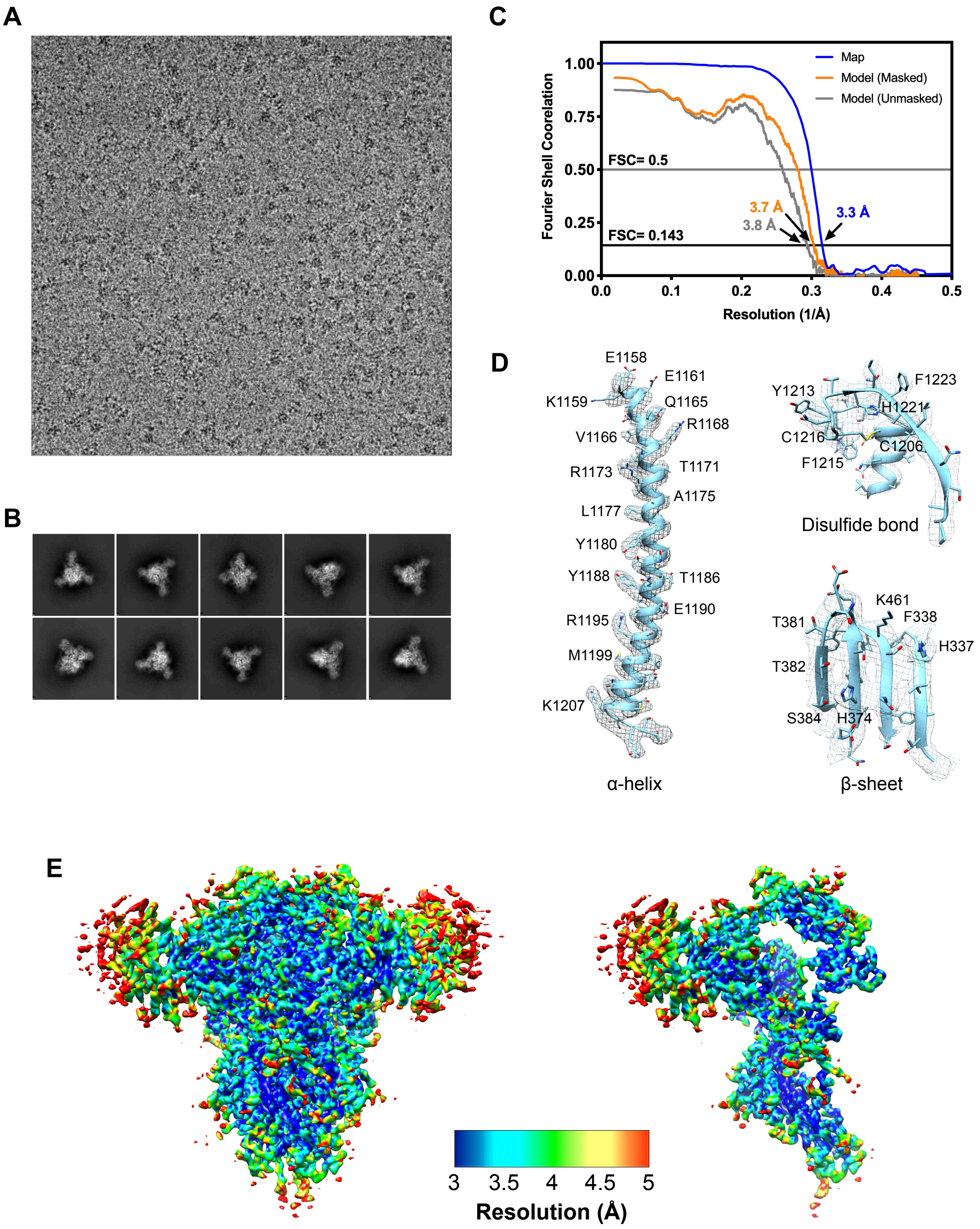

Fig. S3. DPC cryo-EM data analysis and validation. (*A*) Representative DPC cryo-EM micrograph of FIPV-UU4 S protein. (*B*) Selected 2D averaging classes shown in the different orientation. (*C*) Global Fourier shell correlation (FSC) for the FIPV-UU4 S protein and map-to-model FSC analyzed by Phenix. (*D*) Representative EM maps superimposed with the atomic models to highlight the quality of model building. The identities of individual residues are indicated. (*E*) Local resolution map for final EM map analyzed by ResMap.

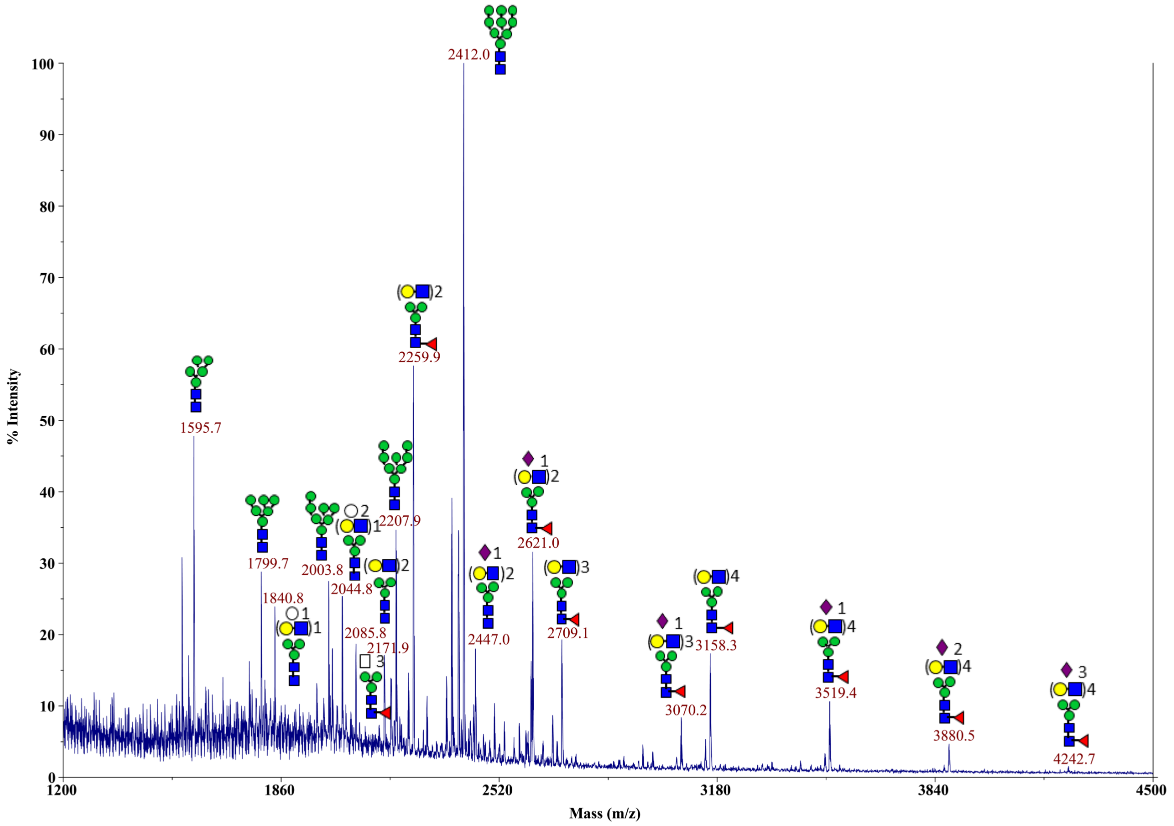

Fig. S4. MALDI-MS profile of reduced, permethylated *N*-glycans released from FIPV-UU4 S protein. Major structures were assigned based on glycosyl composition defined by their molecular masses and the range of commonly expected *N*-glycan structures, without further analysis to determine the possible isomers. Cartoon annotation of the high mannose, hybrid and complex type *N*-glycans were based on the recommended Symbol Nomenclature for Glycans.

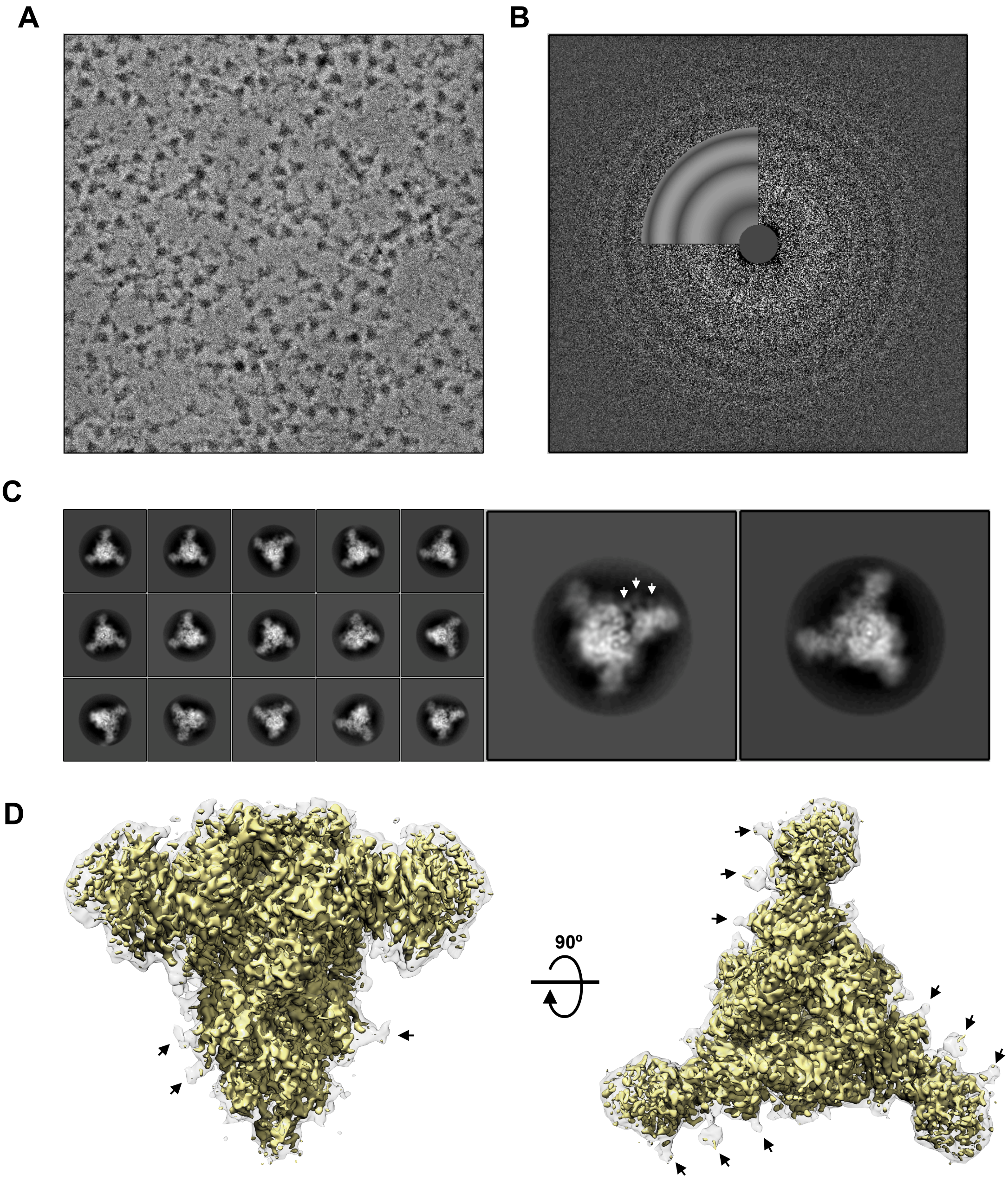

Fig. S5. Cryo-EM micrographs of FIPV-UU4 S protein collected with VPP. (*A*) Representative micrograph a defocus value of 366 nm and a phase shift of 120º. (*B*) Experimental contrast transfer function (CTF) Thon rings superimposed with computed CTF data shown in the upper left quarter. (*C*) The best 2D averaging classes that show additional EM densities corresponding to the putative glycan structures on FIPV-UU4 S protein. (*D*) Superimposition of two 3D maps before (grey, transparent) and after post-processing (yellow). Arrows indicate that glycan density detected in 3D map, which correspond to the same positions shown in (*C*).

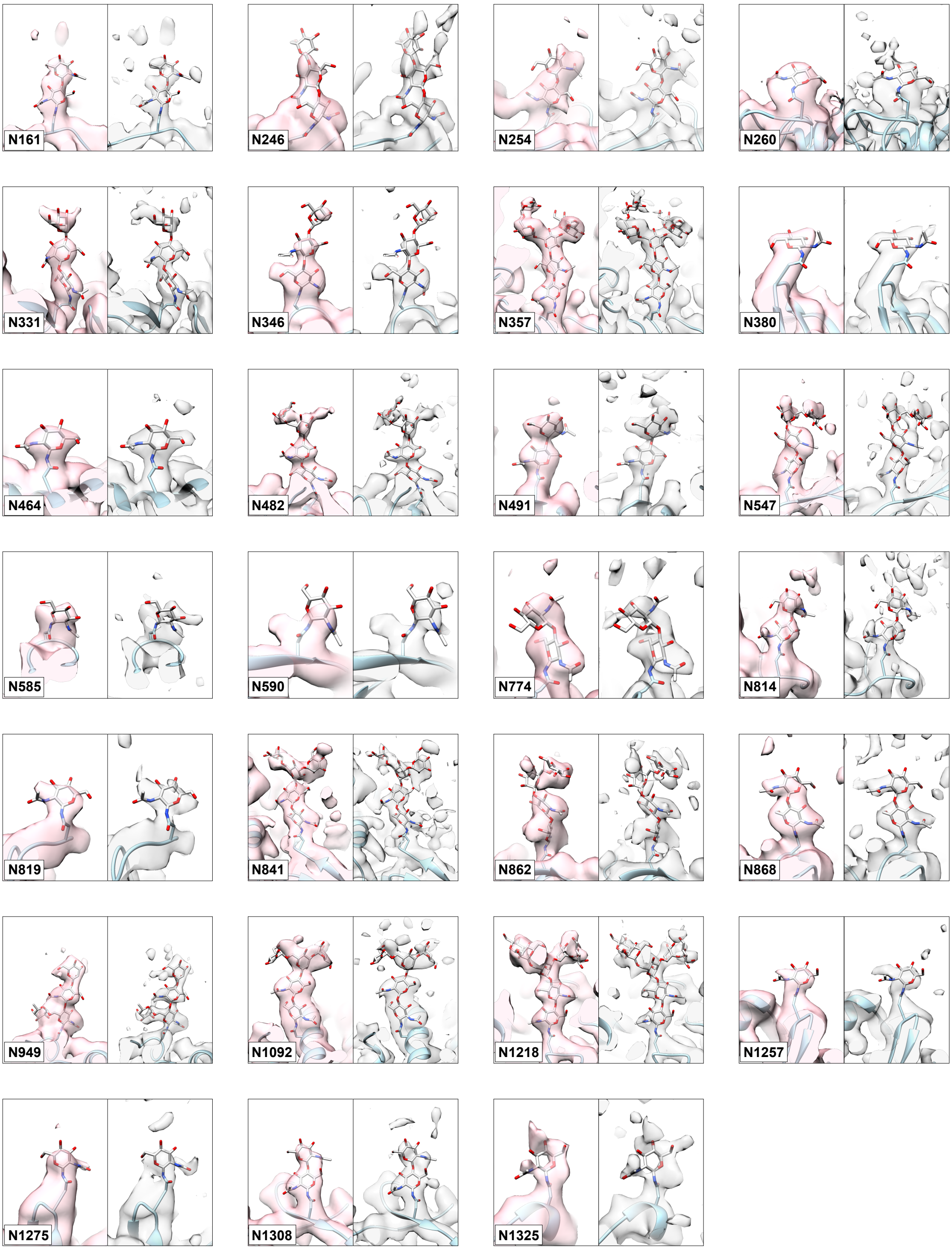

Fig. S6. Gallery of cryo-EM derived *N*-linked glycan densities on the individual asparagine side-chains. The atomic models of individual *N*-linked glycans are shown in a ball-and-stick inside the EM densities. Left panels correspond to the 3.3 Å map without sharpening, whereas the right panels correspond to the map that have been sharpened with a B-factor of -90 $Å^2$.

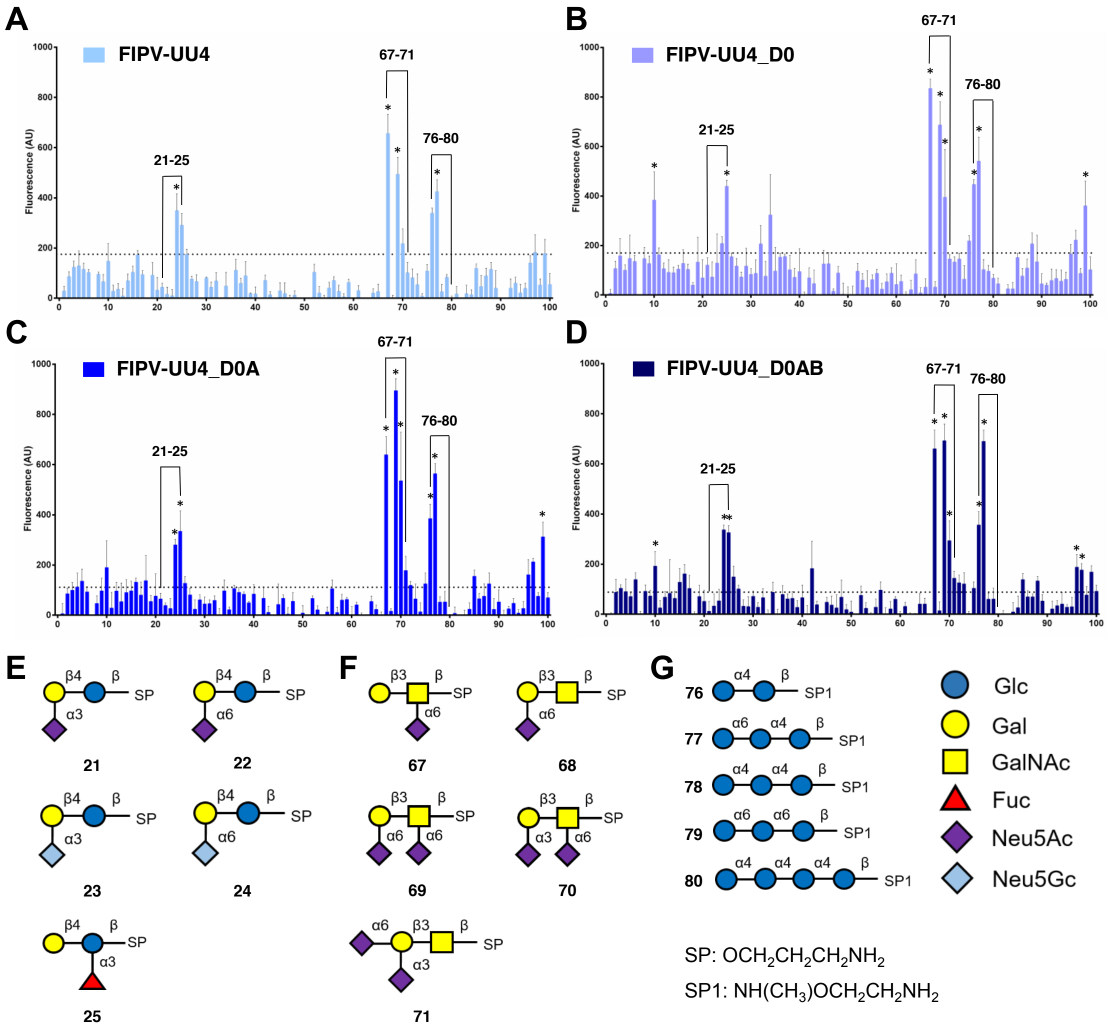

Fig. S7. Glycan array analysis of FIPV-UU4 S protein, (*A*) and its truncated variants, (*B-D*) The amount of binding is reported by the observed fluorescence intensity, which is derived from triplicates with a standard deviation (SD) shown as a vertical line. The dotted line corresponds to the baseline of the array signals, which was derived from the background signals derived from blank measurements. Three groups of glycans that showed significant binding (more than twice the background signals) are indicated with the compound numbers, (*E-G*) and their corresponding glycan structures are shown below with the nomenclatures as defined by the Consortium for Functional Glycomics.

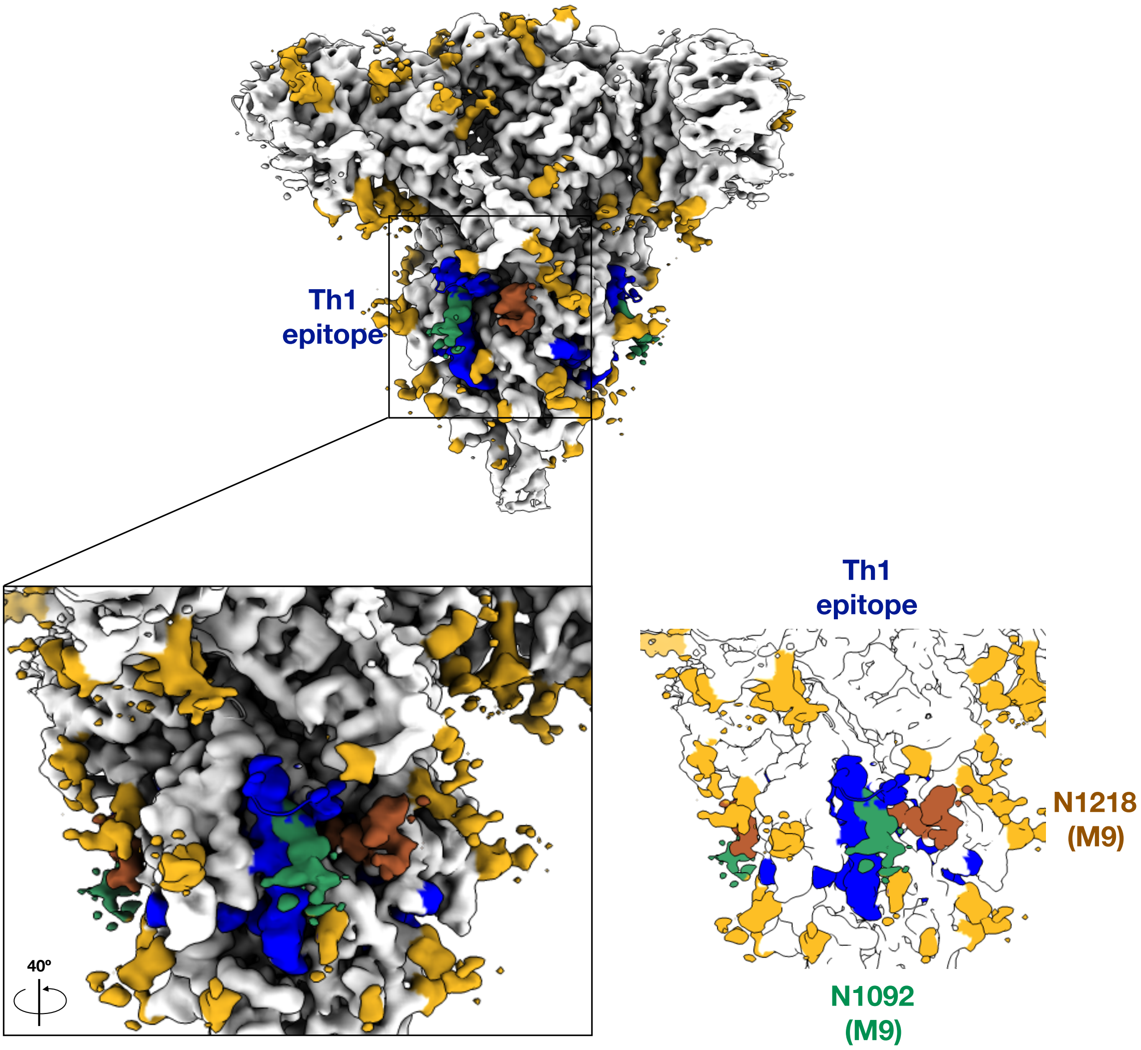

Fig. S8. T-helper 1 (Th1) epitope masked by high-mannose type *N*-glycan (M9) in N1092. The Th1 epitope (residue 1051-1110), which could elicit effective immune responses, is colored in blue. The high-mannose *N*-glycan on N1092 is colored in green and other glycans are colored in gold.

SI Tables

Table S1. List of reported CoV S protein structures and their expression systems and structural details.

**CoV genus**	α		β				γ	δ
**Name**	FIPV-UU4	HCoV-NL63	HCoV-HKU1	SARS	MERS	MHV	IBV	PdCoV
**EM map**	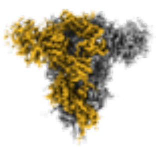	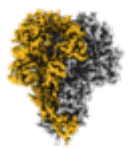	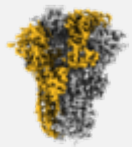	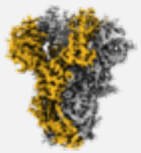	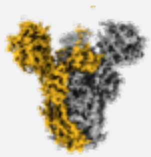	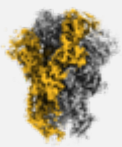	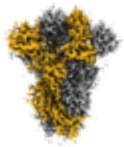	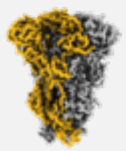
**Host**	Feline (cat)	Human	Human	Human (from palm civet)	Human (from camel)	Murine	Avian	Porcine
**Host receptor**	Unknown	hACE2	Unknown	hACE2	hDPP4	mCEACEM1	aAPN glycan	Unknown
**Expression system**	HEK293	Drosophila S2	HEK293-F	Sf9	Sf9	Drosophila S2	Sf9	Sf9
**Resolution (Å)**	3.3	3.3	4.0	3.2	3.7	4.0	3.9	3.3
**EMDB ID**	EMD-9891	EMD-8331	EMD-8069	EMD-6703	EMD-6704	EMD-6526	EMD-7631	EMD-7063
**PDB ID**	6JX7	5SZS	5I08	5X58	5X59	3JCL	6CV0	6B7N

ACE2: angiotensin-converting enzyme 2; DPP4: dipeptidyl peptidase 4;

CEACAM1: carcinoembryonic antigen-related cell adhesion molecule 1; APN: aminopeptidase

Table S2. FIPV-UU4 S glycopeptides identified by LC-MS/MS.

Residue #	Sequence	Modifications (fixed)	Glycans	Obs. m/z	Calc. m/z	z	ppm err.	Scan Time	Score	PEP 2D	XIC Intensity	Scan #
161	K.IC[+57.02146]HWNPGN[+1216.42286]ISTY.H	C2(Carbamidomethyl / 57.0215)	HexNAc(2)Hex(5)	893.3642	893.3651	3	-1.03	47.13	751.1	1.10E-11	2.02E+07	16247
161	K.IC[+57.02146]HWNPGN[+1565.56014]ISTY.H	C2(Carbamidomethyl / 57.0215)	HexNAc(3)Hex(5)Fuc(1)	1009.7438	1009.7442	3	-0.37	46.58	725.2	1.10E-10	1.51E+07	15925
161	K.IC[+57.02146]HWNPGN[+1768.63952]ISTY.H	C2(Carbamidomethyl / 57.0215)	HexNAc(4)Hex(5)Fuc(1)	1077.4386	1077.4373	3	1.17	46.65	632.2	1.20E-08	1.07E+07	15967
246	Y.IQFVN[+1378.47569]R.T		HexNAc(2)Hex(6)	1077.9605	1077.9622	2	-1.58	23.32	266.9	0.00097	5.69E+08	4387
254	Y.YAYN[+1702.58133]STGGSNY.T		HexNAc(2)Hex(8)	1450.037	1450.0370	2	-0.03	29.62	431.7	2.50E-06	2.32E+05	6862
331	F.VQC[+57.02146]LRPVPTWSN[+1864.63416] NTAVVHF.K	C3(Carbamidomethyl / 57.0215)	HexNAc(2)Hex(9)	1363.9219	1363.9257	3	-2.8	53.32	549	1.00E-05	2.76E+07	19793
346	F.KNDVFC[+57.02146]PN[+1768.63952]VTADV L.R	C6(Carbamidomethyl / 57.0215)	HexNAc(4)Hex(5)Fuc(1)	1120.8088	1120.8108	3	-1.78	54.85	687.7	5.30E-09	7.06E+07	20653
357	F.NLN[+1864.63416]FSDTDVY.T		HexNAc(2)Hex(9)	1526.582	1526.5815	2	0.37	54.38	668.3	8.60E-09	1.73E+06	20381
357	F.NLN[+2026.68698]FSDTDVY.T		HexNAc(2)Hex(10)	1607.6089	1607.6079	2	0.63	54.15	555.6	8.80E-06	1.64E+06	20242
380	F.TFEDN[+1864.63416]TTASITC[+57.02146]Y. S	C12(Carbamidomethyl / 57.0215)	HexNAc(2)Hex(9)	1129.7604	1129.7618	3	-1.23	48.47	627.8	2.30E-08	1.13E+07	17019
392	Y.SSAN[+2059.73493]VTDNQPASGSISHTPF. V		HexNAc(4)Hex(5)Fuc(1)NeuAc(1)	1359.5573	1359.5584	3	-0.85	45.27	686.2	2.40E-09	5.37E+07	15175
392	Y.SSAN[+1768.63952]VTDNQPASGSISHTPF. V		HexNAc(4)Hex(5)Fuc(1)	1262.5265	1262.5266	3	-0.08	38.38	810	2.40E-12	4.75E+07	11275
418	Y.LC[+57.02146]FAN[+1378.47569]F.S	C2(Carbamidomethyl / 57.0215)	HexNAc(2)Hex(6)	1075.4143	1075.4162	2	-1.74	56.45	487.1	1.60E-06	9.70E+07	21622
464	F.SLQPIKSVN[+1864.63416]F.S		HexNAc(2)Hex(9)	1499.1356	1499.1388	2	-2.09	43.18	558.2	2.20E-05	2.35E+07	14012
482	W.TIAYTN[+1864.63416]Y.T		HexNAc(2)Hex(9)	1355.5236	1355.5227	2	0.64	35.28	542.1	1.40E-06	5.17E+06	9616
491	Y.TDVMVDVN[+1864.63416]GTVITR.L		HexNAc(2)Hex(9)	1128.8084	1128.8091	3	-0.62	46.92	664.3	8.60E-09	6.26E+05	16114
547	W.MN[+2059.73493]VTLHVVLNDIEK.K		HexNAc(4)Hex(5)Fuc(1)NeuAc(1)	1228.8695	1228.8741	3	-3.7	82.17	375.9	9.90E-05	7.84E+05	35634
783	F.EFVN[+1768.63952]HTW.S		HexNAc(4)Hex(5)Fuc(1)	1351.0358	1351.0364	2	-0.49	31.37	302	0.0002	2.71E+07	7692
783	F.EFVN[+1216.42286]HTW.S		HexNAc(2)Hex(5)	1074.927	1074.9281	2	-1.03	32.63	271.8	0.00097	2.24E+07	8236
814; 819	K.WNN[+1216.42286]GTSSN[+1216.42286]C[+ 57.02146]TSVITY.S	C9(Carbamidomethyl / 57.0215)	HexNAc(2)Hex(5); HexNAc(2)Hex(5)	1379.5281	1379.5311	3	-2.18	41.15	383.1	8.30E-05	3.85E+06	12861
868	K.N[+1702.58133]FTVAVQAEY.V		HexNAc(2)Hex(8)	1422.5724	1422.5705	2	1.33	56.67	608.8	3.50E-08	5.19E+05	21755
868	K.N[+1864.63416]FTVAVQAEY.V		HexNAc(2)Hex(9)	1503.6001	1503.5969	2	2.12	56.42	693.2	2.90E-08	4.17E+05	21593
949	F.ATVDKFN[+1768.63952]TTAL.G		HexNAc(4)Hex(5)Fuc(1)	1475.1331	1475.1338	2	-0.5	36.43	300.6	0.00043	4.29E+06	10190
949	F.ATVDKFN[+2133.77171]TTAL.G		HexNAc(5)Hex(6)Fuc(1)	1105.4683	1105.4690	3	-0.69	35.88	300.3	0.00027	1.11E+06	9929
1092	F.NNAIGN[+1864.63416]ITLALGK.V		HexNAc(2)Hex(9)	1055.1315	1055.1305	3	0.96	49.82	678.5	3.00E-09	3.19E+07	17775
1218	Y.GFC[+57.02146]GN[+1864.63416]GTHLF.S	C3(Carbamidomethyl / 57.0215)	HexNAc(2)Hex(9)	1487.5666	1487.5623	2	2.89	39.43	454.3	0.00028	1.54E+08	11889
1257	W.SGIC[+57.02146]VN[+2133.77171]DTYAY.L	C4(Carbamidomethyl / 57.0215)	HexNAc(5)Hex(6)Fuc(1)	1132.7742	1132.7740	3	0.14	45.52	516.1	3.20E-06	1.25E+07	15301
1257	W.SGIC[+57.02146]VN[+1768.63952]DTYAY.L	C4(Carbamidomethyl / 57.0215)	HexNAc(4)Hex(5)Fuc(1)	1011.0618	1011.0633	3	-1.43	46.20	339.5	0.00057	1.18E+07	15700
1275	F.SYN[+1622.58161]GTY.M		HexNAc(4)Hex(5)	1163.938	1163.9387	2	-0.65	21.18	285.7	0.00012	1.73E+06	3734
1275	F.SYN[+2133.77171]GTY.M		HexNAc(5)Hex(6)Fuc(1)	946.691	946.6916	3	-0.68	20.57	311.4	0.00072	1.63E+06	3601

1275	F.SYN[+1987.71380]GTY.M		HexNAc(5)Hex(6)	1346.5037	1346.5048	2	-0.88	20.18	301.6	0.00036	1.34E+06	3519
1275	F.SYN[+1768.63952]GTY.M		HexNAc(4)Hex(5)Fuc(1)	1236.9658	1236.9677	2	-1.52	21.57	366.2	0.00023	1.27E+06	3833
1275	F.SYN[+2424.86713]GTY.M		HexNAc(5)Hex(6)Fuc(1)NeuAc(1)	1043.7229	1043.7234	3	-0.51	28.47	304	0.00075	1.26E+06	6281
1275	F.SYN[+1419.50224]GTY.M		HexNAc(3)Hex(5)	1062.3994	1062.3991	2	0.33	21.08	360	8.10E-05	1.25E+06	3712
1275	F.SYN[+1216.42286]GTY.M		HexNAc(2)Hex(5)	960.8589	960.8594	2	-0.5	20.82	404.5	2.80E-06	1.23E+06	3661
1275	F.SYN[+1581.55506]GTY.M		HexNAc(3)Hex(6)	1143.4235	1143.4255	2	-1.76	20.63	324.4	0.0001	1.22E+06	3618
1275	F.SYN[+2133.77171]GTY.M		HexNAc(5)Hex(6)Fuc(1)	1419.5339	1419.5338	2	0.1	20.58	342.6	0.00049	1.04E+06	3606
1275	F.SYN[+2278.80922]GTY.M		HexNAc(5)Hex(6)NeuAc(1)	995.0367	995.0375	3	-0.78	28.45	321.6	0.00064	1.02E+06	6269
1308	F.VQITSC[+57.02146]EVTFLN[+1581.55506]TTHTTF.Q	C6(Carbamidomethyl / 57.0215)	HexNAc(3)Hex(6)	1227.5284	1227.5270	3	1.18	61.15	709.4	3.80E-10	8.80E+05	24390
1325	F.QEIVIDYIDIN[+1540.52851]K.T		HexNAc(2)Hex(7)	1001.7739	1001.7739	3	-0.08	54.77	661.6	1.10E-09	1.41E+07	20610
1325	F.QEIVIDYIDIN[+1378.47569]K.T		HexNAc(2)Hex(6)	947.7553	947.7563	3	-1.14	55.18	948.6	1.10E-14	1.34E+07	20852

N-glycopeptide were digested with trypsin and chymotrypsin. N-Pos. = The aa position of *N*-glycosylation site. Glycans: HexNAc = *N*-acetylhexosamine, Hex = hexose, Fuc = fucose, NeuAc = *N*-acetylneuraminic acid. Green: high-mannose-type glycans ; Blue: hybrid- or complex-type glycans. Cut-off value: Score>200 and PEP2D<0.001. XIC = extracted ion chromatogram.

Table S3. Summary of experimentally verified *N*-glycosylation sites by mass spectrometry and cryo-EM.

Predicted sites	Analytical method			Type of *N*-glycosylation		
	PNGaseF	Glycopeptide	Cryo-EM	High-mannose	Complex	N.D.
**27**						
**31**						
**161**	V	V	V	V	V	
**246**	V	V	V	V		
**254**	V	V*	V	V		
**260**	V		V			V
**331**	V	V	V	V		
**346**	V	V	V		V	
**357**	V	V	V	V		
**380**	V	V	V	V		
**392**	V	V				V
**418**	V	V				V
**464**	V	V	V	V		
**482**		V	V	V		
**491**	V	V*	V	V		
**547**	V	V*	V		V	
**585**			V			V
**590**			V			V
**774**	V		V			V
**783**	V	V		V	V	
**814**	V	V*	V	V		
**819**	V	V*	V	V		
**841**	V		V			V
**862**	V		V			V
**868**	V	V*	V	V		
**949**	V	V*	V		V	
**1092**	V	V	V	V		
**1218**	V	V	V	V		
**1257**	V	V	V	V	V	
**1275**	V	V	V		V	
**1308**		V*	V		V	
**1325**	V	V	V	V		
**1338**	V					V
**1352**						
**1357**						
**1371**	V					V
**1387**	V					V
**Subtotal**	29	24	27	17	8	11
**Total**		33				

Table S4. Multiple structural alignment of FIPV-UU4 S domain 0.

	Z-score	RMSD (Å)	Identity (%)	PDB ID	# of residues used for alignment	CoV genus
**HCoV-NL63**	14.8	2.7	13	5SZS	168	Alphacoronavirus
**BCoV**	11.5	3.7	9	4H14	191	Betacoronavirus
**MHV**	11.1	3.7	9	3JCL	190	Betacoronavirus
**SARS-CoV**	10.8	3.7	11	5X5B	186	Betacoronavirus
**MERS-CoV**	10.6	4,0	8	5X59	190	Betacoronavirus
**Galectin-4**	8.3	3.7	7	4XZP	122	
**Galectin-2**	8.5	3.2	7	1UL9	123	
**Galectin-8**	8.1	3.9	5	4HAN	133	

Table S5. Multiple structural alignment of FIPV-UU4 S domain A.

	Z-score	RMSD (Å)	Identity (%)	PDB ID	# of residues used for alignment	CoV genus
**HCoV-NL63**	19.1	3.1	41	5SZS	228	Alphacoronavirus
**PdCoV**	19.0	2.5	25	6B7N	219	Deltacoronavirus
**IBV**	9.8	3.9	14	6CV0	190	Gammacoronavirus
**SARS-CoV**	9.5	5.9	18	6CS2	194	Betacoronavirus
**MERS-CoV**	8.0	5.8	11	5X59	198	Betacoronavirus
**MHV**	8.0	6.2	12	3JCL	189	Betacoronavirus
**Galectin-4**	8.0	3.7	9	4XZP	114	

Table S6. Multiple structural alignment of FIPV-UU4 S domain B.

	Z-score	RMSD (Å)	Identity (%)	PDB ID	# of residues used for alignment	CoV genus
**HCoV-NL63**	16.0	1.9	23	5SZS	135	Alphacoronavirus
**PdCoV**	13.2	2.3	17	6B7N	124	Deltacoronavirus
**HCoV-229E**	13.2	2.3	22	6ATK	122	Alphacoronavirus
**PRCV**	11.6	2.9	24	4F5C	119	Alphacoronavirus
**IBV**	6.7	3.6	15	6CV0	105	Gammacoronavirus
**HCoV-HKU1**	3.9	3.2	5	5GNB	82	Betacoronavirus
**MERS-CoV**	3.6	4.8	10	5X59	92	Betacoronavirus
**SARS-CoV**	2.8	4.4	5	5XLR	92	Betacoronavirus

Table S7. Multiple structural alignment of FIPV-UU4 S2 subunit.

	Z-score	RMSD (Å)	Identity (%)	PDB ID	# of residues used for alignment	CoV genus
**PdCoV**	39.3	2.2	53	6B7N	497	Deltacoronavirus
**HCoV-NL63**	38.7	3.3	57	5SZS	498	Alphacoronavirus
**SARS-CoV**	29.8	4.0	35	5XLR	427	Betacoronavirus
**MHV**	29.1	3.6	40	3JCL	354	Betacoronavirus
**IBV**	28.1	3.3	34	6CV0	457	Gammacoronavirus
**MERS-CoV**	27.4	4.4	33	5X59	420	Betacoronavirus

SI Movies

Movie S1. Structural overview of FIPV-UU4 S protein.

Movie S2. Representative cryoEM maps for quality assessment.

Movie S3. Mapping *N*-glycosylation of FIPV-UU4 S protein by MS and cryoEM.

References

1. Y. Tao, S. V. Strelkov, V. V. Mesyanzhinov, M. G. Rossmann, Structure of bacteriophage T4 fibritin: a segmented coiled coil and the role of the C-terminal domain. *Structure* **5**, 789-798 (1997).
2. J. Y. Wang *et al.*, Improved expression of secretory and trimeric proteins in mammalian cells via the introduction of a new trimer motif and a mutant of the tPA signal sequence. *Appl Microbiol Biotechnol* **91**, 731-740 (2011).
3. I. Wang, S. Y. Chen, S. T. Hsu, Unraveling the folding mechanism of the smallest knotted protein, MJ0366. *J Phys Chem B* **119**, 4359-4370 (2015).
4. J. Zivanov *et al.*, New tools for automated high-resolution cryo-EM structure determination in RELION-3. *Elife* **7** (2018).
5. T. Grant, A. Rohou, N. Grigorieff, cisTEM, user-friendly software for single-particle image processing. *Elife* **7** (2018).
6. S. Q. Zheng *et al.*, MotionCor2: anisotropic correction of beam-induced motion for improved cryo-electron microscopy. *Nat Methods* **14**, 331-332 (2017).
7. K. Zhang, Gctf: Real-time CTF determination and correction. *J Struct Biol* **193**, 1-12 (2016).
8. A. Kucukelbir, F. J. Sigworth, H. D. Tagare, Quantifying the local resolution of cryo-EM density maps. *Nat Methods* **11**, 63-65 (2014).
9. K. Naydenova, C. J. Russo, Measuring the effects of particle orientation to improve the efficiency of electron cryomicroscopy. *Nat Commun* **8**, 629 (2017).
10. Y. Z. Tan *et al.*, Addressing preferred specimen orientation in single-particle cryo-EM through tilting. *Nature Methods* **14**, 793-+ (2017).
11. A. Punjani, J. L. Rubinstein, D. J. Fleet, M. A. Brubaker, cryoSPARC: algorithms for rapid unsupervised cryo-EM structure determination. *Nat Methods* **14**, 290-296 (2017).
12. P. D. Adams *et al.*, PHENIX: a comprehensive Python-based system for macromolecular structure solution. *Acta Crystallogr D Biol Crystallogr* **66**, 213-221 (2010).
13. P. Emsley, B. Lohkamp, W. G. Scott, K. Cowtan, Features and development of Coot. *Acta Crystallogr D Biol Crystallogr* **66**, 486-501 (2010).
14. A. Waterhouse *et al.*, SWISS-MODEL: homology modelling of protein structures and complexes. *Nucleic Acids Res* **46**, W296-W303 (2018).
15. P. Emsley, M. Crispin, Structural analysis of glycoproteins: building N-linked glycans with Coot. *Acta Crystallogr D Struct Biol* **74**, 256-263 (2018).
16. J. Agirre, Strategies for carbohydrate model building, refinement and validation. *Acta Crystallogr D Struct Biol* **73**, 171-186 (2017).
17. J. Agirre *et al.*, Privateer: software for the conformational validation of carbohydrate

structures. *Nat Struct Mol Biol* **22**, 833-834 (2015).

18. V. B. Chen *et al.*, MolProbity: all-atom structure validation for macromolecular crystallography. *Acta Crystallogr D Biol Crystallogr* **66**, 12-21 (2010).
19. B. A. Barad *et al.*, EMRinger: side chain-directed model and map validation for 3D cryo-electron microscopy. *Nat Methods* **12**, 943-946 (2015).
20. A. Bohne-Lang, C. W. von der Lieth, GlyProt: in silico glycosylation of proteins. *Nucleic Acids Res* **33**, W214-219 (2005).
21. A. Bohne, E. Lang, C. W. von der Lieth, SWEET - WWW-based rapid 3D construction of oligo- and polysaccharides. *Bioinformatics* **15**, 767-768 (1999).
22. A. Bohne, E. Lang, C. W. von der Lieth, W3-SWEET: Carbohydrate modeling by Internet. *J. Mol. Model.* **4**, 33-43 (1998).
23. M. Bohm *et al.*, Glycosciences.DB: an annotated data collection linking glycomics and proteomics data (2018 update). *Nucleic Acids Res* **47**, D1195-D1201 (2019).
24. L. Holm, P. Rosenstrom, Dali server: conservation mapping in 3D. *Nucleic Acids Res* **38**, W545-549 (2010).
25. X. Robert, P. Gouet, Deciphering key features in protein structures with the new ENDscript server. *Nucleic Acids Res* **42**, W320-324 (2014).
26. E. F. Pettersen *et al.*, UCSF Chimera--a visualization system for exploratory research and analysis. *J Comput Chem* **25**, 1605-1612 (2004).
27. T. D. Goddard *et al.*, UCSF ChimeraX: Meeting modern challenges in visualization and analysis. *Protein Sci* **27**, 14-25 (2018).
28. A. Dell *et al.*, Mass spectrometry of carbohydrate-containing biopolymers. *Methods Enzymol* **230**, 108-132 (1994).
29. T. C. Cham *et al.*, Determination of the cell tropism of serotype 1 feline infectious peritonitis virus using the spike affinity histochemistry in paraffin-embedded tissues. *Microbiol Immunol* **61**, 318-327 (2017).
30. C. N. Lin, B. L. Su, C. W. Wu, L. E. Hsieh, L. L. Chueh, Isolation and identification of a novel feline coronavirus from a kitten with naturally occurring feline infectious peritonitis in Taiwan. *Taiwan Vet. J.* **35**, 145-152 (2009).
31. H. J. Hwang, J. W. Han, G. H. Kim, J. W. Han, Functional expression and characterization of the recombinant *N*-acetyl-glucosamine/*N*-acetyl-galactosamine-specific marine algal lectin BPL3. *Mar Drugs* **16** (2018).
32. J. E. McCombs, J. P. Diaz, K. J. Luebke, J. J. Kohler, Glycan specificity of neuraminidases determined in microarray format. *Carbohydr Res* **428**, 31-40 (2016).
